# Supplementary material for: Fetal programming through early weaning shapes the metabotype of Nelore heifers
Source: PLoS One. 2025 Aug 22;20(8):e0327152. doi: 10.1371/journal.pone.0327152 (PMC12373197; doi:10.1371/journal.pone.0327152)
Supplement: Supplementary Table S1 — (DOCX) [file pone.0327152.s001.docx]

Supplementary table S1 – Assignments of the ^1^H NMR spectrum of the metabolites identified in the serum sample in 0.1 mol L^-1^ phosphate buffer. Chemical shifts (in ppm). multiplicity. coupling constants (in Hz) for hydrogens.

| **Metabolites** | **^1^H** |
| --- | --- |
| 3-Hydroxybutyrate | 1.20 d (6.25 Hz); 2.29 dd (14.36 Hz; 6.25 Hz);  2.40 dd (7.10 Hz;14.36 Hz) |
| 3-Hydroxyisovalerate | 1.27 s; 2.36 s |
| 3-Phenylpropionate | 7.38 – 7.34 m; 7.32 – 7.29 m; 7.27 – 7.23 m; 2.91 – 2.85 m; 2.48 – 2.44 m |
| Acetate | 1.90 s |
| Acetoacetate | 2.26 s; 3.42 – 3.46 m |
| Acetone | 2.22 s |
| Alanine | 1.47 d (7.27 Hz); 3.80 – 3.74 m |
| Betaine | 3.25 s; 3.87 – 3.89 m |
| Butyrate | 2.15 – 2.11 m; 1.56 – 1.50 m; 0.90 – 0.86 m |
| Choline | 4.08 – 4.04 m; 3.52 – 3.49 m; 3.19 sl |
| Citrate | 3.51 d (15.15 Hz); 2.65 d (15.15 Hz) |
| Creatine | 3.02 s; 3.92 s |
| Creatine phosphate | 3.03 s; 3.95 s |
| Creatinine | 4.04 s; 3.04 - 3.03 m |
| Formate | 8.44 s |
| Glucose | 4.64 d (7.92 Hz); 3.88 dd (2.13 Hz;13.33 Hz); 3.84 – 3.50 m; 3.74 – 3.69 m; 3.52 dd (3.84 Hz;9.88 Hz);  3.49 t (9.88 Hz); 3.48 – 3.44 m |
| Glutamate | 3.78 – 3.74 m; 2.36 – 2.88 m; 2.16 – 2.08 m; 2.08 – 2.00 m |
| Glutamine | 3.78 – 3.75 m; 2.48 – 2.40 m; 2.18 – 2.10 m |
| Glycine | 3.50 s |
| Glycolate | 3.94 s |
| Hippurate | 7.84 – 7.80 m; 7.65 – 7.61 m; 7.56 – 7.52m; 3.96 – 3.93m |
| Histidine | 7.88 sl; 7.08 sl; 4.00 – 3.96 m |
| Isobutyrate | 2.42 – 2.31 m; 1.06 d (7.05 Hz) |
| Isoleucine | 3.66 d (4.02 Hz); 1.00 d (6.96 Hz); 0.95 – 0.91 m |
| Isopropanol | 4.06 – 3.96 m; 1.16 d (7.04 Hz) |
| Lactate | 4.10 q (6.98; 13.88); 1.32 d (6.98 Hz) |
| Leucine | 1.76 – 1.64 m; 0.95 d (6.15 Hz); 0.94 d (6.15 Hz) |
| Malonate | 3.13 s |
| Methionine | 2.65 – 2.61 m; 2.16 – 2.10 m; 2.12 s |
| N-Nitrosodimethylamine | 3.14 s; 3.82 sl |
| Phenylacetate | 7.38 – 7.33 m; 7.33 – 7.29 m; 3.53 – 3.51 m |
| Phenylalanine | 7.41 m; 7.38 – 7.35 m; 7.33 – 7.30 m |
| Proline | 4.12 – 4.09 m |
| Propionate | 2.20 – 2.15 m; 1.06 – 1.02 m |
| Pyruvate | 2.36 s |
| Sarcosine | 3.60 s; 2.75 s |
| Threonine | 1.32 d (6.50 Hz); 3.58 d (5.15 Hz); 4.26 – 4.21 m |
| Tryptophan | 7.72 d (7.67 Hz); 7.56 – 7.53 m; 7.32 m; 7.28 -7.24 m;  7.21 – 7.18 m; 4.07 – 4.03 m |
| Tyramine | 7.21 – 7.17 m; 6.89 m; 3.27 – 3.22 m; 2.94 – 2.88 m |
| Tyrosine | 7.20 – 7.17 m; 6.91 – 6.88 m |
| Valine | 0.98 d (7.00 Hz); 1.03 d (7.05 Hz); 3.60 d (4.40 Hz) |

s – simplet. d- dublet. m- multiplet.
