# Supplementary material for: Fetal programming through early weaning shapes the metabotype of Nelore heifers
Source: PLoS One. 2025 Aug 22;20(8):e0327152. doi: 10.1371/journal.pone.0327152 (PMC12373197; doi:10.1371/journal.pone.0327152)
Supplement: Supplementary Table S2 — (DOCX) [file pone.0327152.s002.docx]

**Supplementary Table S2. Metabolites concentration in heifers’ serum that didn’t presented the effect of interaction Parit*Treat.**

| **Metabolites** | **PARITY** | |  | **TREATMENT** | |  | **P - value** | | |
| --- | --- | --- | --- | --- | --- | --- | --- | --- | --- |
|  | **Multiparous** | **Second-parity** | **SEM** | **EW** | **CW** | **SEM** | **PARITY** | **TREATMENT** | **PARIT*TREAT** |
| 3-Hydroxybutyrate | 0,2194 | 0,2021 | 0,0189 | 0,2396 | 0,1895 | 0,0189 | 0.5569 | 0.1032 | 0.1465 |
| 3-Hydroxyisovalerate | 0,0769 | 0,0744 | 0,0100 | 0,0850 | 0,0686 | 0,0123 | 0.868 | 0.2888 | 0.9005 |
| 3-Phenylpropionate | 0,0301 | 0,0259 | 0,0039 | 0,0301 | 0,0266 | 0,0034 | 0.4109 | 0.5225 | 0.5183 |
| Acetoacetate | 0,0536 | 0,0544 | 0,0040 | 0,0599 | 0,0494 | 0,0053 | 0.9018 | 0.1483 | 0.511 |
| Acetone | 0,1452 | 0,1073 | 0,0203 | 0,1460 | 0,1130 | 0,0200 | 0.1601 | 0.2564 | 0.3146 |
| Alanine | 0,1494 | 0,1416 | 0,0112 | 0,1671 | 0,1294 | 0,0111 | 0.6963 | 0.0672 . | 0.598 |
| Betaine | 0,1039 | 0,0972 | 0,0077 | 0,1189 | 0,0868 | 0,0087 | 0.6251 | 0.0245 | 0.7327 |
| Butyrate | 0,0260 | 0,0227 | 0,0024 | 0,0283 | 0,0216 | 0,0031 | 0.4123 | 0.1151 | 0.6284 |
| Choline | 0,0081 | 0,0084 | 0,0009 | 0,0102 | 0,0068 | 0,0010 | 0.8432 | 0.0101 | 0.5735 |
| Creatine | 0,0770 | 0,0697 | 0,0075 | 0,0821 | 0,0670 | 0,0087 | 0.5449 | 0.2343 | 0.42 |
| Creatine phosphate | 0,0277 | 0,0225 | 0,0039 | 0,0310 | 0,0209 | 0,0050 | 0.3505 | 0.0861 . | 0.263 |
| Creatinine | 0,0389 | 0,0446 | 0,0078 | 0,0341 | 0,0473 | 0,0075 | 0.6203 | 0.2662 | 0.8374 |
| Formate | 0,1174 | 0,1034 | 0,0066 | 0,1202 | 0,1036 | 0,0062 | 0.197 | 0.1502 | 0.279 |
| Glucose | 1,3876 | 1,2231 | 0,1212 | 1,4826 | 1,1777 | 0,1069 | 0.3549 | 0.1028 | 0.1801 |
| Glutamine | 0,0887 | 0,0616 | 0,0127 | 0,0851 | 0,0689 | 0,0100 | 0.0935 | 0.3706 | 0.0677 . |
| Glycine | 0,1622 | 0,1441 | 0,0125 | 0,1811 | 0,1326 | 0,0161 | 0.4063 | 0.0342 | 0.5767 |
| Glycolate | 0,0658 | 0,0641 | 0,0076 | 0,0645 | 0,0653 | 0,0080 | 0.8861 | 0.9384 | 0.0826 . |
| Histidine | 0,0401 | 0,0317 | 0,0039 | 0,0389 | 0,0341 | 0,0035 | 0.1281 | 0.4409 | 0.0778 . |
| Isobutyrate | 0,0099 | 0,0088 | 0,0008 | 0,0110 | 0,0082 | 0,0008 | 0.373 | 0.0379 | 0.4297 |
| Isoleucine | 0,0722 | 0,0679 | 0,0055 | 0,0817 | 0,0613 | 0,0056 | 0.6649 | 0.0465 | 0.2895 |
| Isopropanol | 0,0345 | 0,0413 | 0,0036 | 0,0397 | 0,0362 | 0,0051 | 0.3141 | 0.5576 | 0.5695 |
| Lactate | 1,0762 | 1,2056 | 0,1199 | 1,3653 | 0,9624 | 0,1461 | 0.6022 | 0.1034 | 0.4362 |
| Leucine | 0,0762 | 0,0701 | 0,0067 | 0,0824 | 0,0663 | 0,0063 | 0.5745 | 0.1507 | 0.4973 |
| Malonate | 0,0377 | 0,0350 | 0,0033 | 0,0423 | 0,0318 | 0,0032 | 0.5695 | 0.0354 | 0.8342 |
| Methionine | 0,0212 | 0,0171 | 0,0018 | 0,0220 | 0,0172 | 0,0016 | 0.1371 | 0.1067 | 0.3123 |
| N-Nitrosodimethylamine | 0,0372 | 0,0367 | 0,0034 | 0,0403 | 0,0343 | 0,0049 | 0.9411 | 0.3418 | 0.1362 |
| Phenylacetate | 0,0223 | 0,0170 | 0,0032 | 0,0267 | 0,0145 | 0,0034 | 0.1683 | 0.003 | 0.53 |
| Phenylalanine | 0,0328 | 0,0256 | 0,0037 | 0,0358 | 0,0245 | 0,0049 | 0.172 | 0.0447 | 0.5005 |
| Proline | 0,2854 | 0,2736 | 0,0724 | 0,3129 | 0,2545 | 0,0360 | 0.8956 | 0.5254 | 0.5572 |
| Propionate | 0,0170 | 0,0152 | 0,0016 | 0,0186 | 0,0143 | 0,0016 | 0.4746 | 0.0878 . | 0.0627 . |
| Pyruvate | 0,0485 | 0,0519 | 0,0065 | 0,0522 | 0,0485 | 0,0069 | 0.728 | 0.6922 | 0.1205 |
| Sarcosine | 0,0131 | 0,0131 | 0,0018 | 0,0154 | 0,0113 | 0,0025 | 0.9963 | 0.1507 | 0.9052 |
| Threonine | 0,0546 | 0,0469 | 0,0081 | 0,0571 | 0,0463 | 0,0050 | 0.4417 | 0.3136 | 0.1961 |
| Tryptophan | 0,0305 | 0,0273 | 0,0035 | 0,0303 | 0,0280 | 0,0038 | 0.528 | 0.6745 | 0.8444 |
| Tyramine | 0,0205 | 0,0181 | 0,0019 | 0,0223 | 0,0171 | 0,0019 | 0.3776 | 0.0665 . | 0.5317 |
| Tyrosine | 0,0281 | 0,0232 | 0,0025 | 0,0286 | 0,0236 | 0,0021 | 0.1994 | 0.2196 | 0.2031 |
| Valine | 0,1585 | 0,1429 | 0,0123 | 0,1709 | 0,1359 | 0,0110 | 0.4464 | 0.1012 | 0.4002 |
