# Supplementary figures and images for: Fetal programming through early weaning shapes the metabotype of Nelore heifers
Source: PLoS One. 2025 Aug 22;20(8):e0327152. doi: 10.1371/journal.pone.0327152 (PMC12373197; doi:10.1371/journal.pone.0327152)

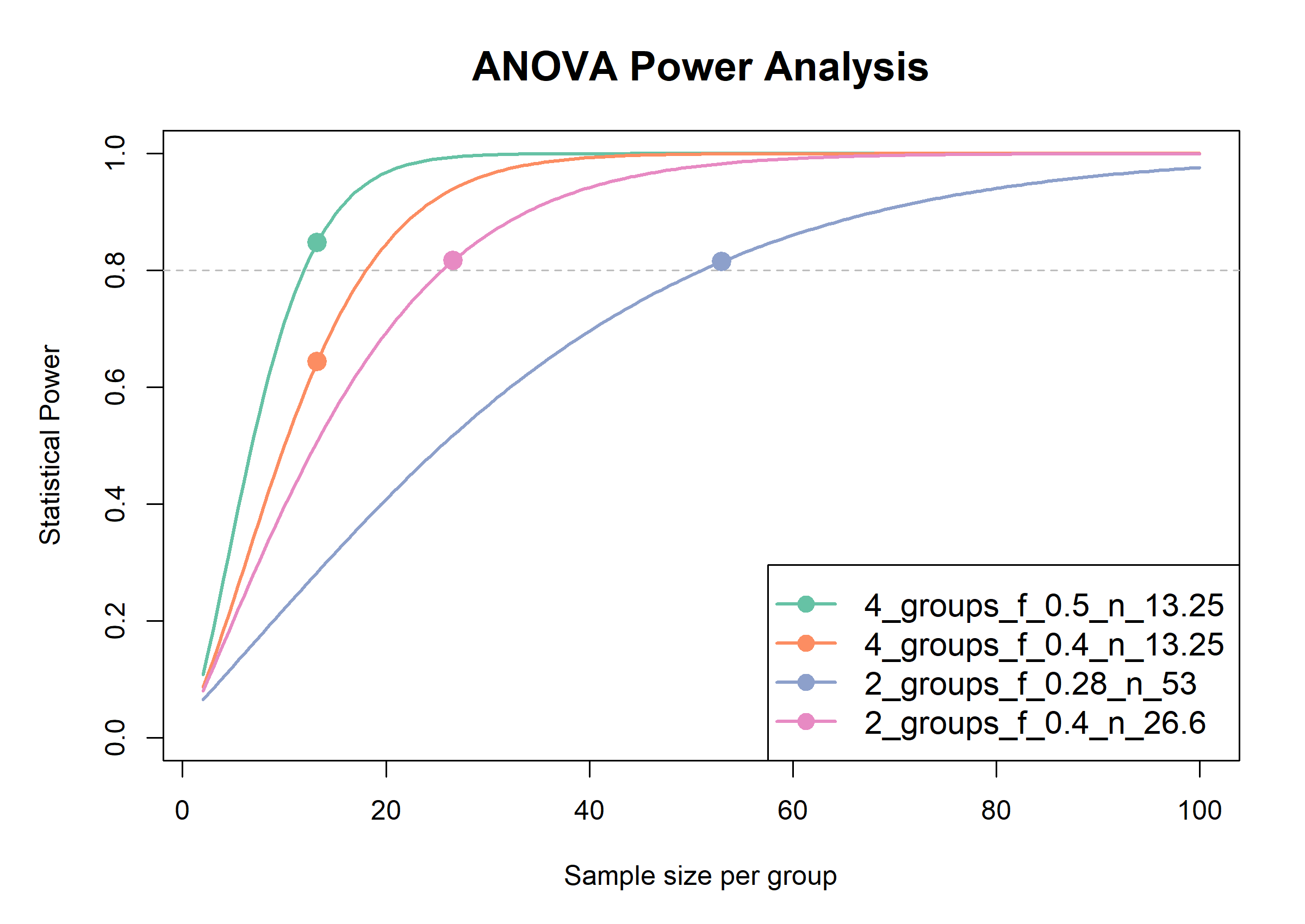

Supplement: Supplementary Figure S1 — (PNG) [file pone.0327152.s004.png]
