# Supplementary material for: Fetal programming through early weaning shapes the metabotype of Nelore heifers
Source: PLoS One. 2025 Aug 22;20(8):e0327152. doi: 10.1371/journal.pone.0327152 (PMC12373197; doi:10.1371/journal.pone.0327152)

PLOS ONE

Fetal Programming through Early Weaning Shapes the Metabotype of Nelore Heifers

--Manuscript Draft--

Manuscript Number:	PONE-D-25-05801
Article Type:	Research Article
Full Title:	Fetal Programming through Early Weaning Shapes the Metabotype of Nelore Heifers
Short Title:	Programmed heifers metabotype
Corresponding Author:	Nara regina brandao consolo Regina Brandão Cônsolo, PhD<br>Universidade de Sao Paulo<br>PIRASSUNUNGA, São Paulo BRAZIL
Keywords:	fetal programming; Metabolism; heifers; metabotype, beef cattle
Abstract:	This study explored the effects of fetal programming induced by early weaning (EW) of the previous calf on Nelore heifers' serum metabolic profile (metabotype). A 2 × 2 factorial design was used, accounting for parity (multiparous vs. secundiparous) and weaning strategy (EW vs. conventional weaning (CW)). A total of 55 female calves were included, comprising 25 born to cows subjected to EW (15 multiparous, 10 secundiparous) and 30 born to cows subjected to CW (15 multiparous, 15 secundiparous). Serum samples were collected from heifers at an average age of 14 months before the timed artificial insemination protocol, and metabolomic profiling was performed using 1H-NMR spectroscopy. Data were analyzed through R software and MetaboAnalyst 6.0. The effects of the weaning strategy and the dam's parity were evaluated using PLS-DA and differential abundance analysis; additionally, enrichment analysis was conducted to identify biological processes linked to the observed changes. The results revealed significant impacts of weaning strategy, dam parity, and their interaction on the metabotype. Heifers from secundiparous cows under EW exhibited elevated concentrations of glutamate, acetate, citrate, and hippurate compared to CW, while no significant differences were observed among weaning strategies in heifers from multiparous cows. Weaning strategies influenced pathways related to energy metabolism, amino acid utilization, and gluconeogenesis. Additionally, heifers born to multiparous cows showed higher levels of phenylalanine, creatine phosphate, and glutamine, suggesting enhanced nutrient availability and metabolic programming compared to those born to secundiparous cows. These findings highlight EW's potential to improve metabolic efficiency and nutrient reallocation, particularly under conditions of limited feed availability or increased physiological demands. This study provides valuable insights into the metabolic programming of beef cattle, supporting strategies to optimize weaning protocols and improve productivity in cattle systems.
Order of Authors:	Anjaleena Yaseen
	Ana Laura dos Santos Munhoz Gôngora
	Thiago Kan Nishimura
	Gabriel Henrique Ribeiro
	Eduardo Solano Pina Santos
	Alanne Tenório Nunes
	Tarique Hussain
	Amjad Hameed
	Luiz Alberto Colnago
	Paulo Roberto Leme
	Arlindo Saran Netto
	Guilherme Pugliesi
	Nara Regina Brandão Cônsolo, PhD

Powered by Editorial Manager® and ProduXion Manager® from Aries Systems Corporation

Additional Information:	
**Question**	**Response**
**Financial Disclosure**<br><br>Enter a financial disclosure statement that describes the sources of funding for the work included in this submission. Review the submission guidelines for detailed requirements. View published research articles from *PLOS ONE* for specific examples.<br><br>This statement is required for submission and **will appear in the published article** if the submission is accepted. Please make sure it is accurate.<br><br>**Funded studies**<br>Enter a statement with the following details:<br>• Initials of the authors who received each award<br>• Grant numbers awarded to each author<br>• The full name of each funder<br>• URL of each funder website<br>• Did the sponsors or funders play any role in the study design, data collection and analysis, decision to publish, or preparation of the manuscript?<br><br>Did you receive funding for this work?	Yes
Please add funding details.<br>as follow-up to "**Financial Disclosure**<br><br>Enter a financial disclosure statement that describes the sources of funding for the work included in this submission. Review the submission guidelines for detailed requirements. View published research articles from *PLOS ONE* for specific examples.<br><br>This statement is required for submission and **will appear in the published article** if the submission is accepted. Please make sure it is accurate.	This study was supported by The Coordenação de Aperfeiçoamento de Pessoal de Nível Superior - Brasil (CAPES) - Finance Codes 001, and Fundação de Amparo à Pesquisa do Estado de São Paulo (FAPESP) grant 2020/08845-3, 2017/18937-0, 2022/07906-4.

**Funded studies**<br>Enter a statement with the following details:<br>• Initials of the authors who received each award<br>• Grant numbers awarded to each author<br>• The full name of each funder<br>• URL of each funder website<br>• Did the sponsors or funders play any role in the study design, data collection and analysis, decision to publish, or preparation of the manuscript?<br><br>Did you receive funding for this work?"	
Please select the country of your main research funder (please select carefully as in some cases this is used in fee calculation).<br>as follow-up to "**Financial Disclosure**<br><br>Enter a financial disclosure statement that describes the sources of funding for the work included in this submission. Review the submission guidelines for detailed requirements. View published research articles from *PLOS ONE* for specific examples.<br><br>This statement is required for submission and **will appear in the published article** if the submission is accepted. Please make sure it is accurate.<br><br>**Funded studies**<br>Enter a statement with the following details:<br>• Initials of the authors who received each award<br>• Grant numbers awarded to each author<br>• The full name of each funder<br>• URL of each funder website<br>• Did the sponsors or funders play any role in the study design, data collection and analysis, decision to publish, or preparation of the manuscript?<br><br>Did you receive funding for this work?"	BRAZIL - BR
**Competing Interests**	The authors have declared that no competing interests exist.

Use the instructions below to enter a competing interest statement for this submission. On behalf of all authors, disclose any competing interests that could be perceived to bias this work—acknowledging all financial support and any other relevant financial or non-financial competing interests.<br><br>This statement is required for submission and **will appear in the published article** if the submission is accepted. Please make sure it is accurate and that any funding sources listed in your Funding Information later in the submission form are also declared in your Financial Disclosure statement.<br><br>View published research articles from *PLOS ONE* for specific examples.<br><br>**NO authors have competing interests**<br><br>Enter: *The authors have declared that no competing interests exist.*<br><br>**Authors with competing interests**<br><br>Enter competing interest details beginning with this statement:<br><br>*I have read the journal's policy and the authors of this manuscript have the following competing interests: [insert competing interests here]*<br><br>* typeset	
**Ethics Statement**<br><br>Enter an ethics statement for this submission. This statement is required if the study involved:<br><br>• Human participants<br>• Human specimens or tissue<br>• Vertebrate animals or cephalopods<br>• Vertebrate embryos or tissues	The experiment was approved by the Ethics Committee of the School of Animal Science and Food Engineering of the University of São Paulo, under the protocol number 2884250620

• Field research<br><br>Write "N/A" if the submission does not require an ethics statement.<br><br>General guidance is provided below. Consult the submission guidelines for detailed instructions. **Make sure that all information entered here is included in the Methods section of the manuscript.**	

Powered by Editorial Manager® and ProduXion Manager® from Aries Systems Corporation

**Format for specific study types**<br><br>**Human Subject Research (involving human participants and/or tissue)**<br>• Give the name of the institutional review board or ethics committee that approved the study<br>• Include the approval number and/or a statement indicating approval of this research<br>• Indicate the form of consent obtained (written/oral) or the reason that consent was not obtained (e.g. the data were analyzed anonymously)<br><br>**Animal Research (involving vertebrate animals, embryos or tissues)**<br>• Provide the name of the Institutional Animal Care and Use Committee (IACUC) or other relevant ethics board that reviewed the study protocol, and indicate whether they approved this research or granted a formal waiver of ethical approval<br>• Include an approval number if one was obtained<br>• If the study involved *non-human primates*, add *additional details* about animal welfare and steps taken to ameliorate suffering<br>• If anesthesia, euthanasia, or any kind of animal sacrifice is part of the study, include briefly which substances and/or methods were applied<br><br>**Field Research**<br><br>Include the following details if this study involves the collection of plant, animal, or other materials from a natural setting:<br>• Field permit number<br>• Name of the institution or relevant body that granted permission	
**Data Availability**<br><br>Authors are required to make all data underlying the findings described fully available, without restriction, and from the time of publication. PLOS allows rare exceptions to address legal and ethical concerns. See the PLOS Data Policy and FAQ for detailed information.	Yes - all data are fully available without restriction

A Data Availability Statement describing where the data can be found is required at submission. Your answers to this question constitute the Data Availability Statement and **will be published in the article**, if accepted.<br><br>**Important:** Stating 'data available on request from the author' is not sufficient. If your data are only available upon request, select 'No' for the first question and explain your exceptional situation in the text box.<br><br>Do the authors confirm that all data underlying the findings described in their manuscript are fully available without restriction?	
**Describe where the data may be found in full sentences. If you are copying our sample text, replace any instances of XXX with the appropriate details.**<br><br>• If the data are **held or will be held in a public repository**, include URLs, accession numbers or DOIs. If this information will only be available after acceptance, indicate this by ticking the box below. For example: *All XXX files are available from the XXX database (accession number(s) XXX, XXX.).*<br>• If the data are all contained **within the manuscript and/or Supporting Information files**, enter the following: *All relevant data are within the manuscript and its Supporting Information files.*<br>• If neither of these applies but you are able to provide **details of access elsewhere**, with or without limitations, please do so. For example:<br><br>*Data cannot be shared publicly because of [XXX]. Data are available from the XXX Institutional Data Access / Ethics Committee (contact via XXX) for researchers who meet the criteria for access to confidential data.*<br><br>*The data underlying the results presented in the study are available from (include the name of the third party*	Data can be shared once required to the corresponding author

and contact information or URL).<br>• This text is appropriate if the data are owned by a third party and authors do not have permission to share the data.<br><br>* typeset	
Additional data availability information:	

Click here to access/download;Manuscript;Metabolomics and Fetal-programming_SUBMITTED.docx

Fetal Programming through Early Weaning Shapes the Metabotype of Nelore Heifers

Anjaleena Yaseen[1,5]¶, Ana Laura dos Santos Munhoz Gôngora[2]¶, Thiago Kan Nishimura[3]¶, Gabriel Henrique Ribeiro[4]¶, Eduardo Solano Pina Santos[5]¶, Alanne Tenório Nunes[6]¶, Tarique Hussain[1]¶, Amjad Hameed[1]¶, Luiz Alberto Colnago[4]¶, Paulo Roberto Leme, Arlindo Saran Netto, Guilherme Pugliesi[3]¶, Nara Regina Brandão Cônsolo[2]¶*

[1]Animal Sciences Division, Nuclear Institute for Agriculture and Biology College, Pakistan Institute of Engineering and Applied Sciences, Faisalabad, Pakistan

[2] Department of Nutrition and Animal Production, School of Veterinary Medicine and Animal Science, University of São Paulo, Pirassununga, SP, Brazil.

[3] Department of Animal Reproduction, School of Veterinary Medicine and Animal Science, University of São Paulo, Pirassununga, SP, Brazil.

[4] Embrapa Instrumentação Agropecuária, São Carlos, Brazil.

[5] Department of Animal Science, College of Animal Science and Food Engineering, University of São Paulo, Pirassununga, SP, Brazil

[6] Department of Veterinary Medicine, College of Animal Science and Food Engineering, University of São Paulo, Pirassununga, SP, Brazil

* Corresponding author

nara.consolo@usp.br (NRBC, 0000-0002-8389-7794)

Abstract

This study explored the effects of fetal programming induced by early weaning (EW) of the previous calf on Nelore heifers' serum metabolic profile (metabotype). A 2 × 2 factorial design was used, accounting for parity (multiparous vs. secundiparous) and weaning strategy (EW vs. conventional weaning (CW)). A total of 55 female calves were included, comprising 25 born to cows subjected to EW (15 multiparous, 10 secundiparous) and 30 born to cows subjected to CW (15 multiparous, 15 secundiparous). Serum samples were collected from heifers at an average age of 14 months before the timed artificial insemination protocol, and metabolomic profiling was performed using ^{1}H-NMR spectroscopy. Data were analyzed through R software and MetaboAnalyst 6.0. The effects of the weaning strategy and the dam's parity were evaluated using PLS-DA and differential abundance analysis; additionally, enrichment analysis was conducted to identify biological processes linked to the observed changes. The results revealed significant impacts of weaning strategy, dam parity, and their interaction on the metabotype. Heifers from secundiparous cows under EW exhibited elevated concentrations of glutamate, acetate, citrate, and hippurate compared to CW, while no significant differences were observed among weaning strategies in heifers from multiparous cows. Weaning strategies influenced pathways related to energy metabolism, amino acid utilization, and gluconeogenesis. Additionally, heifers born to multiparous cows showed higher levels of phenylalanine, creatine phosphate, and glutamine, suggesting enhanced nutrient availability and metabolic programming compared to those born to secundiparous cows. These findings highlight EW's potential to improve metabolic efficiency and nutrient reallocation, particularly under conditions of limited feed availability or increased physiological demands. This study provides valuable insights into the metabolic programming of beef cattle, supporting strategies to optimize weaning protocols and improve productivity in cattle systems.

1. Introduction

Fetal developmental programming is the complex interplay between fetal development and long-term physiological outcomes [1]. This concept is grounded in the idea that the maternal environment during critical periods of development can profoundly and permanently shape an individual's physiology and metabolism. Prenatal organogenesis and tissue differentiation are intricately influenced by maternal nutrition, metabolic processes, and environmental factors [2,3]. In response to these maternal stimuli, the fetus undergoes adaptive cellular, biochemical, and molecular adjustments, mediated by cellular metabolism and placental function [4,5]. These adaptations influence prenatal growth and maturation and have far-reaching effects on reproductive potential, skeletal muscle development, meat quality, and postnatal physiological functions [6,7].

Beef cattle production in tropical regions of Brazil uses, in general, *Bos indicus* breeds and faces significant challenges during the dry season due to a sharp decline in forage quality and availability [8]. Prolonged droughts, especially in late summer and autumn, result in feed scarcity and low-quality forage, which fail to meet the nutritional demands of gestating beef cows and their developing fetuses [8,9]. Additionally, as weaning typically occurs at around 8 months of age in *Bos indicus* cows (Baruselli et al., 2018), these lactating cows require increased energy and nutrients to support milk production [11] during the rapid growth phase of the fetus (Bauman and Bruce Currie, 1980). Also, primiparous cows have higher energy requirements than multiparous cows because they need extra nutrients to sustain their body growth (NRC, 2016). This nutritional stress negatively impacts fetal development and can have lasting effects on offspring [10]. When these heightened demands are unmet, competition for nutrients arises between milk production and fetal development, potentially impairing reproductive growth and pubertal development in the offspring [7,11,12].

In this line of reasoning, anticipating the weaning from 8 to 5 months in *Bos indicus* females can positively impact pregnancy outcomes by reducing the nutritional demands on lactating cows

during the dry season. This practice reallocates nutrients to support fetal growth and development, promoting long-term improvements in offspring health, metabolism, productivity, and reproductive performance in sustainable beef cattle production systems in tropical and subtropical regions [13–15]. In this sense, understanding the metabolism of programmed animals can provide insights into how the process influences and/or differentiates the phenotype according to fetal programming by early weaning of the previous calf.

Metabolic imprinting, driven by genetics, epigenetics, and environmental factors, is fundamental in shaping an individual's metabolic profile or metabotype [16]. This process influences key aspects of animal metabolism, including genetic potential, the onset of puberty, and overall productivity and reproductive performance [17–19]. Early weaning within a fetal programming framework provides an opportunity to investigate metabolic pathways that influence the long-term metabolic state and reproductive development of Nelore heifers [20]. These insights can guide the development of weaning strategies, nutritional supplementation, and management practices to enhance herd health and productivity. However, the relationship between fetal programming, the age at weaning of the previous calf, and the metabolism of puberty in heifers remains unexplored.

This study hypothesizes that the metabotype of heifers is influenced by fetal programming by the early weaning of the previous calf and it can be a function of the cow's parity order. To test this hypothesis, a metabolomics approach was used to examine the impact of fetal programming through early weaning of the previous calf on the metabotype of heifers before the timed artificial insemination protocol. By analyzing these metabolic profiles, this study aims to gain deeper insights into how early weaning may influence fetal programming, particularly regarding underlying metabolic mechanisms, and if there are any differences in heifers' metabotype according to the cow's parity order. These findings could help guide the development of more effective management strategies aimed at improving reproductive performance and overall productivity in beef cattle systems.

2. Methodology

2.1. Ethics statement

The experiment was approved by the Ethics Committee of the School of Animal Science and Food Engineering of the University of São Paulo, under the protocol number 2884250620. The study was carried out at the campus of Pirassununga (SP) of the University of São Paulo, Brazil.

2.2. Experimental design, animals and management

This study employed a 2 × 2 factorial design with two main factors: parity (secundiparous or multiparous) and weaning strategy (early weaning, EW, at 5 months age; or conventional weaning, CW at 8 months age). A total of 208 cows (57 primiparous and 151 multiparous at the beginning of the experiment) were included. These cows calved in 2019 and became pregnant through timed artificial insemination (TAI) during the 2020 breeding season (November 2020 to January 2021). Animals were randomly assigned to one of two weaning strategies: 1) EW, where calves were weaned at approximately 150 days of age (mean ± SEM: 149 ± 2.0 days), including 27 primiparous and 74 multiparous cows; and 2) CW, where calves were weaned at approximately 240 days of age (mean ± SEM: 247 ± 2.4 days), including 27 primiparous and 77 multiparous cows. During the 2021 calving season (September to October), a total of 55 female Nellore calves were included in this study. Of these, 25 calves were born to cows subjected to early weaning (15 from multiparous cows and 10 from secundiparous cows), and 30 calves were born to cows subjected to conventional weaning (15 from multiparous cows and 15 from secundiparous cows). The calves were kept with the cows on *Brachiaria*

spp. pasture, with unrestricted access to water, until weaning at 240 days of age. Animals' management and diets followed as described by Nishimura et al. [21–24].

2.3. Blood sample collection

Blood samples (approximately 10 mL) were collected via jugular venipuncture using evacuated tubes (BD Vacutainer, São Paulo, Brazil) without anticoagulant, immediately before the initiation of the TAI protocol when the heifers were 14 months of old (13.5 ± 0.11 months). Samples were centrifuged at 2,800 × g for 15 minutes at 4°C. The supernatant serum was transferred into labeled plastic tubes and stored at -80°C until analysis.

2.4. Metabolites extraction

A double extraction protocol was performed to isolate serum metabolites for NMR analysis, as described by Da Costa et al. [25]. Initially, 500 µL of serum was transferred into 1.5-mL conical tubes, and 400 µL of ice-cold methanol and 400 µL of ice-cold acetone were added, followed by vortexing for 10 seconds. Then, 400 µL of ice-cold chloroform was added, and the samples were vortexed again for 10 seconds. The samples were then placed on ice for 10 minutes before being centrifuged at 10,000 × g for 10 minutes at 4°C to precipitate proteins. This extraction process was repeated to enhance metabolite recovery. For the second round, 550 µL (approximately) of the supernatant was transferred to a new 2 mL Eppendorf tube. Ice-cold methanol (300 µL) and acetone (300 µL) were added, followed by vortexing for 10 seconds. Next, 300 µL of ice-cold chloroform was introduced, and the samples were vortexed again for 10 seconds. After 10 minutes on ice, the samples underwent centrifugation at

10,000 × g for 10 minutes at 4°C to remove residual proteins. Finally, approximately 1 mL of the supernatant was transferred to a new 2 mL conical tube for pooling and subsequent analysis.

2.5. NMR spectroscopy and spectral processing

One-dimensional (1D) ^{1}H-NMR spectroscopy was utilized for metabolite profiling, with the analyses conducted at EMBRAPA Instrumentation (São Carlos, São Paulo, Brazil), as described by Cônsolo et al. [17]. Briefly, proton NMR spectra were acquired at 300 K on a Bruker Avance 14.1 T spectrometer (Bruker Corporation, Karlsruhe, Baden-Wurttemberg, Germany) at 600.13 MHz for ^{1}H. A BBO 5-mm probe was used for sample analysis, with D_2O serving as the lock solvent and DSS as the chemical shift reference for ^{1}H. Standard 1D proton NMR spectra were acquired using a single 90° pulse experiment, with each spectrum consisting of the sum of 64 free induction decays. Water suppression was achieved using the Bruker "zgesgp" pulse sequence (excitation sculpting with gradients). Key acquisition parameters included a 90° pulse duration of 13.05 ms, a recycle delay of 0.5 s, 64k data points, 128 scans, an acquisition time of 3.89 s, and a spectral width of 10.03 ppm.

2.6. Metabolite identification and quantification

^{1}H-NMR spectra were processed using Chenomx NMR Suite Professional 7.7 software (Chenomx Inc., Edmonton, Canada). The spectra were referenced to the DSS methyl peak at 0.00 ppm, which was also used as a resolution indicator, i.e. as an internal standard for quantification. Metabolites quantification in serum spectra was conducted using the Profiler module of Chenomx NMR Suite Professional software with an in-built 1D spectral library (https://hmdb.ca/). Quantification was

performed by comparing the area of selected metabolite peaks to the area under the DSS methyl peak, which had a known concentration of 0.5 mM in all samples. The metabolite concentration data was transferred to Excel, where the sample identifiers were subsequently incorporated.

2.7. Statistical analysis

Statistical analyses were conducted using the R software. First, the normality of residuals and the homogeneity of variances were verified using the UNIVARIATE procedure. Metabolite data were analyzed in a completely randomized design using the MIXED procedure, considering the fixed effect of dam's parity order (multiparous and secundiparous), weaning strategy (EW or CW), and their interaction, and the animal was considered the experimental unit. The means were compared by the Student's t-test. Significance was declared at $p < 0.05$.

The metabolomic data were also analyzed using MetaboAnalyst 6.0 (http://www.metaboanalyst.ca/). Before analysis, data were preprocessing, including log transformation and Pareto scaling. Differential abundance analysis was conducted to identify metabolites with significant differences between groups, with p-values adjusted through false discovery rate (FDR) correction. Volcano plots were then generated to visualize these findings. Metabolites with FDR-adjusted p-values < 0.05 were considered significant. Enrichment analysis was performed on the significant metabolites, with a threshold of $p < 0.1$, to identify biological processes or pathways associated with the observed metabolic changes.

3. Results

3.1. Serum metabolites profile

Thirty-nine metabolites were quantified in the ^{1}H-NMR spectrum of blood serum. The identified metabolites included non-essential amino acids, essential amino acids, biogenic amines, organic acids, sugars, and vitamins. The influence of dam's parity order, weaning strategies, and their interaction on metabolite concentrations in serum samples was assessed. The metabolites that exhibited significant differences among the groups are summarized in Table 1. A significant interaction between parity and weaning strategies was observed for glutamate ($p < 0.01$), as well as acetate, citrate, and hippurate ($p < 0.05$). Specifically, the early weaning (EW) strategy was associated with an increase in the concentrations of these metabolites compared to the conventional weaning (CW) in secundiparous cows, whereas no significant effect was observed on metabolite concentrations for the different weaning strategies in multiparous cows.

Table 1. Effects of dam's parity order and weaning strategy on metabolite concentration (mmol) in serum samples of Nelore (*Bos indicus*) heifers at 14 months of old.

	Multiparous		Secundiparous		*P*-value
	EW[1]	CW[2]	EW[1]	CW[2]	PAR*WE[3]
Acetate	0.5016 [a]	0.608 [a]	0.6617 [a]	0.3898 [b]	0.026 *
Citrate	0.0658 [a]	0.0719 [a]	0.089 [a]	0.0534 [b]	0.029 *
Glutamate	0.1507 [ab]	0.1804 [a]	0.2143 [a]	0.1034 [b]	0.009 **
Hippurate	0.0353 [ab]	0.034 [ab]	0.0464 [a]	0.022 [b]	0.045 *

[1]Earling weaning; [2]Conventional weaning; [3]Interaction between parity and weaning strategy.

The PLS-DA analysis was used to visualize the differences in metabolite profiles between the EW and CW treatments and between the dam's parity order (multiparous vs. secundiparous). The component 1/component 2 scores plot (Figs 1 and 2) shows the CW and EW treatments as well as the parity categories overlap.

Fig 1. Partial least-squares discriminant analysis (PLS-DA) of serum metabolite distribution according to conventional weaning (CW, at 8 months age) and early weaning (EW, at 5 months age). There were two significant Components which together explain 62% of the total variance (Component 1 58% and Component 2 4%). The distribution data presented a partial overlap in the serum metabolome data according to the treatments, suggesting differences in serum metabolome for heifers according to weaning strategies.

Fig 2. Partial least-squares discriminant analysis (PLS-DA) of serum metabolite distribution according to dam's category, as multiparous (MUL) and secundiparous (SEC). There were two significant Components which together explain 63% of the total variance (Component 1 56% and Component 2 7%). The distribution data presented a partial overlapping in the serum metabolome data according to the categories, suggesting differences in serum metabolome for heifers according to the dam's parity order.

To further investigate the influence of weaning strategies and dam's parity order on the serum metabolome profile of heifers, differential expression analysis was performed using a t-test, with P-values adjusted through false discovery rate (FDR) correction. The results of these analyses were visualized using volcano plots (Figs 3 and 4). For the weaning strategy comparison, the EW group exhibited significantly higher concentrations ($p < 0.05$) of valine, alanine, lactate, methionine, arginine, hippurate, isoleucine, and creatine phosphate compared to the CW group (Fig 3). Regarding dams' parity, heifers born to multiparous cows demonstrated a metabolome characterized by higher concentrations of phenylalanine, creatine phosphate, glutamine, glutamate, and 3-hydroxy isovalerate compared to those born to secundiparous cows (Fig 4).

Fig 3. Volcano plot illustrating the differential expression of serum metabolites in heifers subjected to early weaning (EW, at 5 months age) compared to conventional weaning (CW, at 8 months age) strategies. Metabolites with significantly higher concentrations in the early weaning (EW) group ($p < 0.05$) include valine, alanine, lactate, methionine, arginine, hippurate, isoleucine, and creatine phosphate.

Fig 4. Volcano plot showing the differential expression of serum metabolites in heifers born to multiparous cows compared to those born to secundiparous cows. Metabolites with significantly higher concentrations in heifers from multiparous cows ($p < 0.05$) include phenylalanine, creatine phosphate, glutamine, glutamate, and 3-hydroxyisovalerate.

3.2. Pathway analysis

For the pairwise comparison of weaning strategies, significant differences ($p < 0.05$) were observed in multiple metabolic pathways, including selenoamino acid metabolism, urea cycle, arginine and proline metabolism, valine, leucine, and isoleucine degradation, spermidine and spermine biosynthesis, glycine and serine metabolism, propanoate metabolism, pyruvate metabolism, glutathione metabolism, glutamate metabolism, alanine metabolism, gluconeogenesis, tryptophan metabolism, and betaine metabolism (Fig 5). These findings highlight the diverse metabolic alterations induced by early weaning compared to conventional weaning. In the comparison of heifers from multiparous and secundiparous cows, the most relevant pathways ($p < 0.1$) were nicotinate and nicotinamide metabolism, phenylalanine and tyrosine metabolism, pyrimidine metabolism, lysine degradation, arachidonic acid metabolism, and malate-aspartate shuttle (Fig 6). These results suggest that the metabolic profile of heifers is influenced by dam parity, potentially reflecting differences in nutrient allocation and developmental processes.

Fig 5. Enrichment analysis of metabolite sets comparing early and conventional weaning strategies. Significant pathways ($p < 0.05$) include selenoamino acid metabolism, urea cycle, arginine and proline metabolism, valine, leucine, and isoleucine degradation, spermidine and spermine biosynthesis, glycine and serine metabolism, propanoate metabolism, pyruvate metabolism, glutathione metabolism, glutamate metabolism, alanine metabolism, gluconeogenesis, tryptophan metabolism, and betaine metabolism.

Fig 6. Enrichment analysis of metabolite sets comparing heifers from multiparous and secundiparous cows. Significant pathways ($p < 0.1$) include nicotinate and nicotinamide metabolism, phenylalanine and tyrosine metabolism, pyrimidine metabolism, lysine degradation, arachidonic acid metabolism, and malate-aspartate shuttle.

4. Discussion

This study hypothesized that the metabotype of heifers is influenced by fetal programming, triggered by the early weaning (at 5 months age) of the previous calf. The findings confirm this hypothesis, revealing significant differences in the metabotypes of heifers from different weaning

strategy groups at 14 months of age. Additionally, the metabotype varied based on dams' parity order, and an interaction between parity and weaning strategy was observed, where the most pronounced differences between the weaning strategies occurred in heifers from secundiparous cows.

In heifers born to secundiparous cows, the EW strategy was associated with increased concentrations of key metabolites, including glutamate, acetate, citrate, and hippurate, compared to CW. In contrast, no significant effects on metabolite concentrations were observed in heifers from multiparous cows under the same weaning strategies. This disparity may be attributed to the differing physiological demands and adaptive capacities of the two groups. Multiparous cows, having completed their body growth, can efficiently allocate nutrients to fetal development, whereas secundiparous cows may still face the dual challenge of supporting their own continued growth alongside fetal nutrient demands [26,27]. As a result, heifers from secundiparous cows may benefit more from the early weaning of their previous calf than heifers from primiparous cows, due the energetic status of the dams.

This data is corroborated by Nishimura et al. [22], who evaluated the same animals and explained that early weaning significantly improved the body condition score, body weight, average daily gain, and carcass fat deposition in cows during the 90 days they were without calves. The authors attribute these results to a reduction in the metabolic demands causing of the dam due to the cessation of lactation, causing a positively affects the dam's metabolic and nutritional status [22]. This is particularly beneficial for second-parity cows, as they are still in a developmental stage.

The elevated metabolites observed in heifers from secundiparous cows under EW, such as acetate, citrate, glutamate, and hippurate, highlight critical metabolic adjustments. These metabolites play critical roles in energy metabolism and nutrient utilization. Acetate, for instance, is a key substrate for fatty acid synthesis and energy production, particularly important during periods of elevated energy demand [28]. Citrate serves as an intermediate in the tricarboxylic acid cycle, contributing to cellular energy generation, which is essential for supporting growth and metabolic adaptations [29]. Similarly, glutamate, a key amino acid, is involved in protein synthesis and serves as a critical energy source for

rapidly growing tissues [30]. Finally, hippurate, a metabolite derived from gut microbial fermentation, may reflect alterations in nutrient absorption and energy reallocation [31].

The observed differences suggest that EW may heighten metabolic demands in secundiparous cows, leading to increased mobilization of these metabolites to sustain both maternal and fetal requirements, as previously discussed above and supported by Nishimura et al. [22]. This aligns with findings that younger cows are more susceptible to metabolic stress and negative energy balance (NEB) during critical periods [32–34]. Primiparous cows, for example, require greater energy for simultaneous growth and reproductive functions, which can prolong recovery from NEB and delay ovulation [32,33]. Additionally, differences in feed intake, endocrine responses, and blood metabolite levels between primiparous and multiparous cows during transition periods further emphasize their differing metabolic challenges [27,35]. Importantly, the lack of differences in metabolite concentrations between the weaning strategies in multiparous cows suggests that these animals have a greater ability to maintain metabolic stability, likely due to their more mature physiological status and reduced growth-related energy demands. These findings underscore that the impact of early weaning on metabolic programming is particularly significant in younger, still-developing cows.

Furthermore, the comparison of heifers born to multiparous and secundiparous cows revealed notable differences in metabolic profiles. Heifers from multiparous cows exhibited higher concentrations of metabolites such as phenylalanine, creatine phosphate, glutamine, and glutamate, which are essential for energy reserves, amino acid mobilization, and nitrogen transport [30,36]. These observations point to a more favorable uterine environment in multiparous cows, enabling enhanced nutrient availability during fetal development. This aligns with findings from previous studies with the same animals [23,24] which showed that heifers born to multiparous cows had were heavier from 13 to 15 months age, presented a higher body score condition, greater carcass fat deposition and lower serum IGF-1 concentration than from than heifers from SEC cows, showing a better metabolic status. Which resulted in a more appropriate status for puberty and reproduction. Nishimura et al. [23] also reported that

heifers from multiparous dams presented a higher number of cumulus-oocyte complexes recovered, which were heavier than those collected from heifers born to secundiparous cows. Pathway analysis in the present study further supports these observations, with significant differences identified in nicotinate and nicotinamide metabolism, phenylalanine and tyrosine metabolism, and pyrimidine metabolism, among others. These pathways, critical for energy production, amino acid utilization, and cellular growth, highlight the developmental advantages of heifers born to multiparous cows, whereas the metabolic challenges in secundiparous cows likely reduce nutrient availability for the developing fetus [37–39].

When comparing the metabotype of heifers considering the weaning strategy, it was observed that the EW group exhibited significantly higher concentrations of valine, alanine, lactate, methionine, arginine, hippurate, isoleucine, and creatine phosphate compared to the CW group. These metabolites are involved in key metabolic pathways enriched in this study, including urea cycle, branched-chain amino acid (BCAA) metabolism, gluconeogenesis, pyruvate metabolism, arginine and proline metabolism, and glutathione metabolism. Notably, similar metabolic pathways, such as the glucose-alanine cycle, urea cycle, and glutathione metabolism, were enriched in Nellore heifers receiving creep feeding supplementation, which also improved their reproductive performance [40]. This alignment underscores the critical metabolic shifts induced by enhanced nutrient availability, whether through the metabolic programming observed in the present study or the supplementation provided by creep feeding. These findings highlight the vital role of early nutritional interventions in enriching essential metabolic pathways, ultimately promoting growth, metabolic adaptation, and improved reproductive outcomes.

The elevated concentrations of valine and isoleucine, two essential BCAAs, are particularly noteworthy due to their roles in rumen microbial protein synthesis – accounting for up to 90% of amino acids reaching the small intestine in cattle [41] – and as key substrates for energy production under metabolic stress. And it may reflect enhanced mobilization of amino acids to fuel gluconeogenesis and

energy metabolism for EW heifers. The increase in alanine further underscores these shifts in amino acid and energy metabolism, since alanine is synthesized from pyruvate and BCAAs in muscle tissue and accounts for a significant proportion of amino acid nitrogen transported to the liver in ruminants, playing a central role in nitrogen transport and gluconeogenesis [42].

Similarly, the higher levels of lactate in the EW group align with enhanced pyruvate metabolism and gluconeogenesis, indicating a shift toward anaerobic glycolysis. Lactate, produced as a by-product of pyruvate under anaerobic conditions, is a crucial gluconeogenic substrate in the liver [43]. The observed increase in creatine phosphate provides additional evidence of enhanced muscle energy metabolism in heifers programmed. As a rapid reservoir of high-energy phosphates, creatine phosphate plays a pivotal role in regenerating ATP during periods of high energy demand, particularly through the action of creatine kinase [44]. The higher concentrations of arginine and methionine in the EW group are also significant in the context of altered amino acid metabolism. Arginine is central to urea cycle function and serves as a precursor for nitric oxide synthesis, which is critical for vascular regulation and immune responses [45]. Meanwhile, methionine plays a key role in glutathione metabolism and methylation reactions through its derivative, S-adenosylmethionine [46].

Putting together, this data my suggests an increased reliance on amino acids and glycolytic intermediates to maintain glucose homeostasis for programed heifers, thereby supporting energy demands under conditions of altered nutrient partitioning. Additionally, the EW group may have a upregulation of antioxidant defense mechanisms and detoxification processes, especially important during oxidative stress.

Despite these significant metabolic differences, the reproductive performance, body development and hepatic gene expresison of these animals was not notably impacted in a parallel study [23,24]. This lack of phenotypic impact may be explained by the optimal nutritional conditions provided during the study, which likely mitigated the physiological consequences of EW. In environments where forage quality or feed nutritive value is limited during mid-to-late lactation, these

metabolic differences may translate into more pronounced phenotypic effects, particularly in younger, still-growing cows [26].

Several studies have shown that lactation significantly increases maintenance energy requirements in cows [47–49]. In this context, EW can lead to a notable reduction in the cow's maintenance energy costs by terminating milk production and shifting nutrient priorities. The energy savings from reduced maternal maintenance and lactation demands can be redirected to enhance calf growth, either through additional feed or improved nutrient utilization efficiency [50]. Our findings suggest that EW is particularly beneficial in nutrient-limited systems, where it alleviates the trade-off lactating cows face between milk production and supporting fetal or post-weaning growth, improving metabolic efficiency and system sustainability by reallocating energy to the fetus. Therefore, EW strategies should be evaluated within the context of environmental and nutritional factors, as their benefits may be more pronounced under limited feed availability conditions or higher metabolic and energy demands.

5. Conclusion

This study demonstrated that the metabotype of heifers is influenced by fetal programming through weaning strategy, as well as by dams' parity, with early weaning having a greater impact on secundiparous cows due to their ongoing growth demands. The observed interaction between parity and weaning strategy highlights the heightened metabolic challenges faced by younger dams. Our novel findings emphasize the overall benefits of early weaning strategies for fetal programming to enhance programed heifers' nutrient utilization efficiency, particularly in challenging conditions such as limited feed availability or periods of higher physiological demand of their dams.

6. References

[1] Harvey KM, Cooke RF, Moriel P. Impacts of Nutritional Management During Early Postnatal Life on Long-Term Physiological and Productive Responses of Beef Cattle. Frontiers in Animal Science 2021;2:730356. https://doi.org/10.3389/FANIM.2021.730356/BIBTEX.

[2] Srinivasan M, Patel MS. Metabolic programming in the immediate postnatal period. Trends in Endocrinology and Metabolism 2008;19:146–52. https://doi.org/10.1016/j.tem.2007.12.001.

[3] Patel MS, Srinivasan M. Metabolic Programming in the Immediate Postnatal Life. Ann Nutr Metab 2011;58:18. https://doi.org/10.1159/000328040.

[4] Miller DR, Blache D, Jackson RB, Downie EF, Roche JR. Metabolic maturity at birth and neonate lamb survival: Association among maternal factors, litter size, lamb birth weight, and plasma metabolic and endocrine factors on survival and behavior. J Anim Sci 2010;88:581–92. https://doi.org/10.2527/JAS.2009-2170.

[5] Dunlap KA, Brown JD, Keith AB, Satterfield MC. Factors controlling nutrient availability to the developing fetus in ruminants. J Anim Sci Biotechnol 2015;6:1–10. https://doi.org/10.1186/S40104-015-0012-5/FIGURES/3.

[6] Bell AW. Prenatal programming of postnatal productivity and health of livestock: a brief review. Aust J Exp Agric 2006;46:725–32. https://doi.org/10.1071/EA06006.

[7] Funston RN, Larson DM, Vonnahme KA. Effects of maternal nutrition on conceptus growth and offspring performance: Implications for beef cattle production. J Anim Sci 2010;88:E205–15. https://doi.org/10.2527/JAS.2009-2351.

[8] Souza NRD, Junqueira TL, Cavalett O. Opportunities and challenges for bioenergy-livestock integrated systems in Brazil. Ind Crops Prod 2021;173:114091. https://doi.org/10.1016/J.INDCROP.2021.114091.

[9] Underwood KR, Tong JF, Price PL, Roberts AJ, Grings EE, Hess BW, et al. Nutrition during mid to late gestation affects growth, adipose tissue deposition, and tenderness in cross-bred beef steers. Meat Sci 2010;86:588–93. https://doi.org/10.1016/J.MEATSCI.2010.04.008.

[10] Nääs I de A, Romanini CEB, Salgado DDA, Lima KAO, do Vale MM, Labigalini MR, et al. Impact of global warming on beef cattle production cost in Brazil. Sci Agric 2010;67:01–8. https://doi.org/10.1590/S0103-90162010000100001.

[11] McGuffey RK. A 100-Year Review: Metabolic modifiers in dairy cattle nutrition. J Dairy Sci 2017;100:10113–42. https://doi.org/10.3168/JDS.2017-12987.

[12] Nwachukwu CU. Review article: Nutritional effects on fetal development during gestation in ruminants. Nigerian Journal of Animal Production 2020;47:245–56. https://doi.org/10.51791/NJAP.V47I2.184.

[13] Baldwin RL, McLeod KR, Klotz JL, Heitmann RN. Rumen Development, Intestinal Growth and Hepatic Metabolism In The Pre- and Postweaning Ruminant. J Dairy Sci 2004;87:E55–65. https://doi.org/10.3168/JDS.S0022-0302(04)70061-2.

[14] da Silva JH, Bondan C, González FHD. Metabolic Profile of Holstein Cows: Reference Values in Herds of Rio Grande do Sul - Brazil. Acta Sci Vet 2022;50. https://doi.org/10.22456/1679-9216.120192.

[15] de Lima RS, Martins T, Lemes KM, Binelli M, Madureira EH. Effect of a puberty induction protocol based on injectable long acting progesterone on pregnancy success of beef heifers serviced by TAI. Theriogenology 2020;154:128–34. https://doi.org/10.1016/J.THERIOGENOLOGY.2020.05.036.

[16] Reis MM, Cooke RF, Cappellozza BI, Marques RS, Guarnieri Filho TA, Rodrigues MC, et al. Creep-feeding to stimulate metabolic imprinting in nursing beef heifers: impacts on heifer

growth, reproductive and physiological variables. Animal 2015;9:1500–8. https://doi.org/10.1017/S1751731115000828.
[17] Cônsolo NRB, Silva J, Buarque VM, Barbosa LC, H. Padilla A, Colnago LA, et al. Metabolomic signature of genetic potential for muscularity in beef cattle. Anim Biotechnol 2022;33:1308–17. https://doi.org/10.1080/10495398.2021.1894164.
[18] Diskin MG, Mackey DR, Roche JF, Sreenan JM. Effects of nutrition and metabolic status on circulating hormones and ovarian follicle development in cattle. Anim Reprod Sci 2003;78:345–70. https://doi.org/10.1016/S0378-4320(03)00099-X.
[19] Sargentini C, Bozzi R, Rivera PD, Giorgetti A, Martini A, Lupi P, et al. Onset of puberty in Maremmana heifers. Ital J Anim Sci 2007;6:385–94. https://doi.org/10.4081/IJAS.2007.385.
[20] Schubach KM, Smith SB, Washburn KE. Impacts of Gestational and Early-Life Nutrition on Productive and Health Responses of Beef Cattle. Texas A&M University, 2020.
[21] Nishimura TK, Goulart RS, Ramírez-Zamudio GD, da Silva AG, de Paula Carlis MS, Abitante G, et al. Effects of early weaning on productive and reproductive performance of Nelore heifers. J Anim Sci 2023;101. https://doi.org/10.1093/JAS/SKAD379.
[22] Nishimura TK, da Silva AG, Abitante G, Dahlen CR, Goulart RS, Zamudio GDR, et al. Effects of early weaning on the reproductive performance of suckled Nelore cows in the subsequent breeding season. J Anim Sci 2023;101:skad330. https://doi.org/10.1093/JAS/SKAD330.
[23] Nishimura TK, Locoselli L, Teixeira NA, Bridi A, Silveira JC, Zamudio GDR, et al. Can early weaning in suckled beef cows affect the fetal programming of the next generation? II. Effects on puberty and reproductive performance. Not Published 2024.
[24] Nishimura TK, Carlis MSP, Matos ACD, Feltrin IR, Goulart RS, Zamudio GDR, et al. Can early weaning in suckled beef cows affect the fetal programming of the next generation? I. Effects on the body development. Not Published 2024.
[25] da Costa MB, Cônsolo NRB, Silva J, Buarque VLM, Padilla ARH, Coutinho ID, et al. Performance, carcass traits and serum metabolomic profile of Nellore males with different genetic potential for post-weaning growth. Animal 2020;14:873–80. https://doi.org/10.1017/S1751731119002520.
[26] Wiseman A, Redden M, McGee A, Spencer C, Reuter R, Horn G, et al. Effects of timing of weaning on energy utilization in primiparous beef cows and post-weaning performance of their progeny1. J Anim Sci 2019;97:1198–211. https://doi.org/10.1093/JAS/SKZ019.
[27] Wathes DC, Cheng Z, Bourne N, Taylor VJ, Coffey MP, Brotherstone S. Differences between primiparous and multiparous dairy cows in the inter-relationships between metabolic traits, milk yield and body condition score in the periparturient period. Domest Anim Endocrinol 2007;33:203–25. https://doi.org/10.1016/J.DOMANIEND.2006.05.004.
[28] Forsberg NE, Baldwin RL, Smith NE. Roles of Acetate and Its Interactions with Glucose and Lactate in Cow Mammary Tissue. J Dairy Sci 1984;67:2247–54. https://doi.org/10.3168/jds.S0022-0302(84)81572-6.
[29] Iacobazzi V, Infantino V. Citrate-new functions for an old metabolite. Biol Chem 2014;395:387–99. https://doi.org/10.1515/HSZ-2013-0271/MACHINEREADABLECITATION/RIS.
[30] Holeček M. Roles of malate and aspartate in gluconeogenesis in various physiological and pathological states. Metabolism 2023;145. https://doi.org/10.1016/J.METABOL.2023.155614/ATTACHMENT/B53E8192-4232-48DC-83A5-63579CB1A8DB/MMC1.DOCX.
[31] Lees HJ, Swann JR, Wilson ID, Nicholson JK, Holmes E. Hippurate: The natural history of a mammalian-microbial cometabolite. J Proteome Res 2013;12:1527–46. https://doi.org/10.1021/PR300900B/ASSET/IMAGES/LARGE/PR-2012-00900B_0004.JPEG.

[32] Jasari B, Atanasov B, Celeska I, Radeski M, Nikolovski M, Dovenski T. Metabolic profile and hormonal status comparison between primiparous and multiparous non-cyclic cows. Maced Vet Rev 2018;41:195–201. https://doi.org/10.2478/MACVETREV-2018-0022.
[33] Lucy MC, Staples CR, Thatcher WW, Erickson PS, Cleale RM, Firkins JL, et al. Influence of diet composition, dry-matter intake, milk production and energy balance on time of post-partum ovulation and fertility in dairy cows. Animal Science 1992;54:323–31. https://doi.org/10.1017/S0003356100020778.
[34] Civiero M, Cabezas-Garcia EH, Ribeiro-Filho HMN, Gordon AW, Ferris CP. Relationships between energy balance during early lactation and cow performance, blood metabolites, and fertility: A meta-analysis of individual cow data. J Dairy Sci 2021;104:7233–51. https://doi.org/10.3168/JDS.2020-19607/ASSET/1D17B1CA-9CB8-4220-B1D2-7E105B6A9A25/MAIN.ASSETS/GR3.JPG.
[35] Macmillan K, Hayirli A, Doepel L, Dyck BL, Subramaniam E, Ambrose DJ, et al. Interrelationships among plasma metabolites, production, and ovarian follicular function in dairy cows. Can J Anim Sci 2018;98:631–41. https://doi.org/10.1139/CJAS-2017-0182/ASSET/IMAGES/LARGE/CJAS-2017-0182F5.JPEG.
[36] Funk C, Clark A, Connett RJ. How Phosphocreatine Buffers Cyclic Changes in ATP Demand in Working Muscle. Adv Exp Med Biol 1989;248:687–92. https://doi.org/10.1007/978-1-4684-5643-1_76.
[37] Gilbreath KR, Bazer FW, Satterfield MC, Wu G. Amino Acid Nutrition and Reproductive Performance in Ruminants. Adv Exp Med Biol 2021;1285:43–61. https://doi.org/10.1007/978-3-030-54462-1_4/TABLES/2.
[38] Wang Y, Wu H, Zhang Y, Fei M, Li Z, Ren D, et al. Nicotinamide benefited amino acid metabolism and rumen fermentation pattern to improve growth performance of growing lambs. Anim Biosci 2024;37:1913–22. https://doi.org/10.5713/AB.24.0015.
[39] Ferreira MF de L, Rennó LN, Rodrigues II, Detmann E, Paulino MF, de Campos Valadares Filho S, et al. Effects of parity order on performance, metabolic, and hormonal parameters of grazing beef cows during pre-calving and lactation periods. BMC Vet Res 2021;17:1–15. https://doi.org/10.1186/S12917-021-03019-0/TABLES/4.
[40] Catussi BLC, Ferreira JR, Lo Turco EG, Morgulis SCF, Baruselli PS. Metabolic imprinting in beef calves supplemented with creep feeding on performance, reproductive efficiency and metabolome profile. Sci Rep 2024;14. https://doi.org/10.1038/s41598-024-60216-1.
[41] Flint HJ, Bayer EA. Plant Cell Wall Breakdown by Anaerobic Microorganisms from the Mammalian Digestive Tract. Ann N Y Acad Sci 2008;1125:280–8. https://doi.org/10.1196/ANNALS.1419.022.
[42] Reynolds CK, Tyrrell HF. Effects of mesenteric vein L-alanine infusion on liver metabolism in beef heifers fed on diets differing in forage:concentrate ratio. Br J Nutr 1991;66:437–50. https://doi.org/10.1079/BJN19910046.
[43] Baird GD, Walt JG Van Der, Bergman EN. Whole-body metabolism of glucose and lactate in productive sheep and cows. British Journal of Nutrition 1983;50:249–65. https://doi.org/10.1079/BJN19830094.
[44] Casey A, Greenhaff PL. Does dietary creatine supplementation play a role in skeletal muscle metabolism and performance?1234. Am J Clin Nutr 2000;72:607S-617S. https://doi.org/10.1093/AJCN/72.2.607S.
[45] Wu G, Bazer FW, Davis TA, Kim SW, Li P, Marc Rhoads J, et al. Arginine metabolism and nutrition in growth, health and disease. Amino Acids 2008 37:1 2008;37:153–68. https://doi.org/10.1007/S00726-008-0210-Y.
[46] Wu G. Amino acids: metabolism, functions, and nutrition. Amino Acids 2009 37:1 2009;37:1–17. https://doi.org/10.1007/S00726-009-0269-0.

[47] Patle BR, Mudgal VD. Utilization of dietary energy for maintenance, milk production and lipogenesis by lactating crossbred cows during their midstage of lactation. British Journal of Nutrition 1977;37:23–33. https://doi.org/10.1079/BJN19770004.
[48] Patle BR, Mudgal VD. Maintenance requirements for energy in cross-bred cattle*. British Journal of Nutrition 1975;33:127–39. https://doi.org/10.1079/BJN19750018.
[49] Ferrell CL, Jenkins TG. Cow Type and the Nutritional Environment: Nutritional Aspects. J Anim Sci 1985;61:725–41. https://doi.org/10.2527/JAS1985.613725X.
[50] Sciences NA of, Earth D on. Nutrient requirements of beef cattle 2016.

 Click here to access/download;Figure;Figure1.png

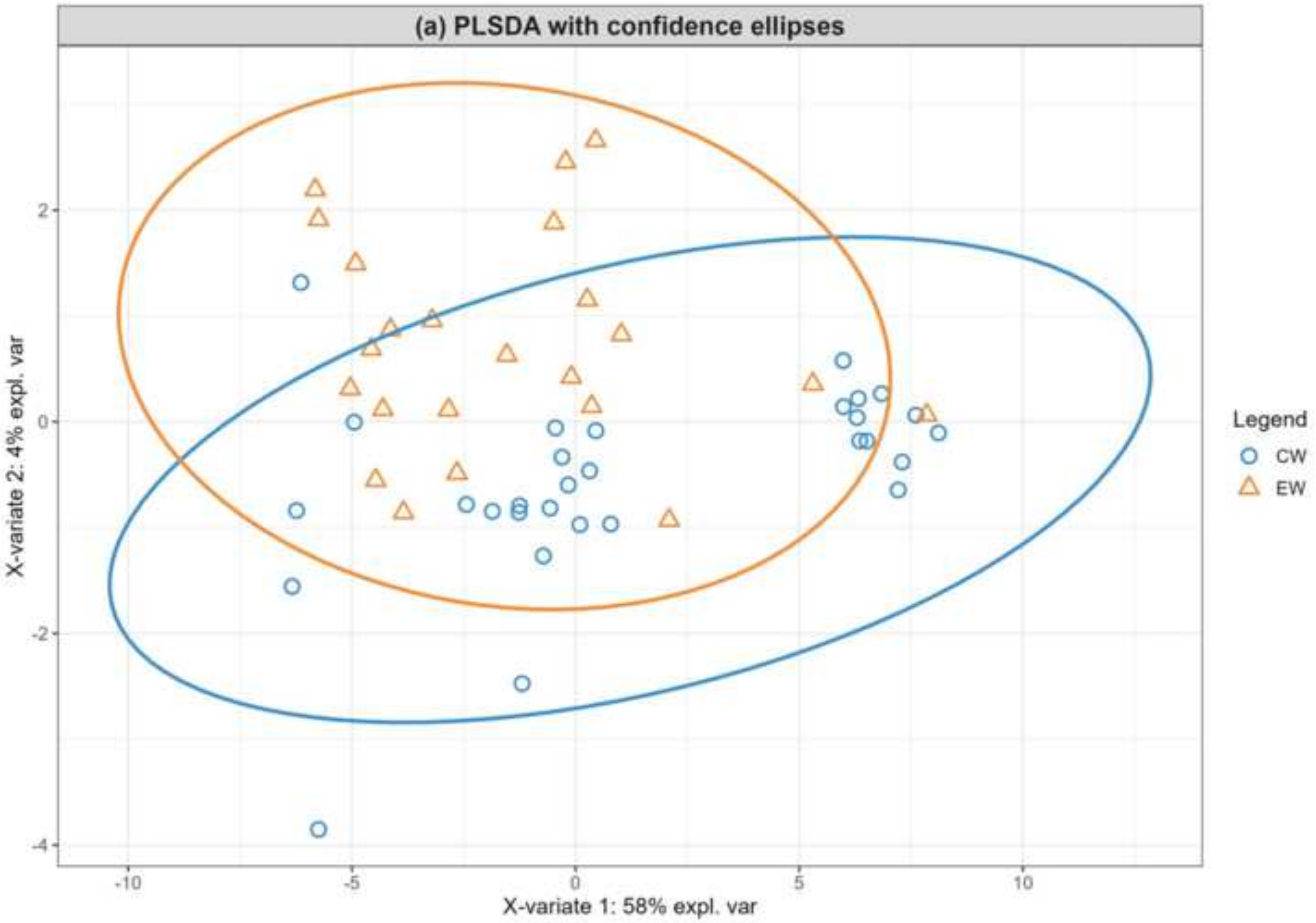

Figure2

Click here to access/download;Figure;Figure2.png

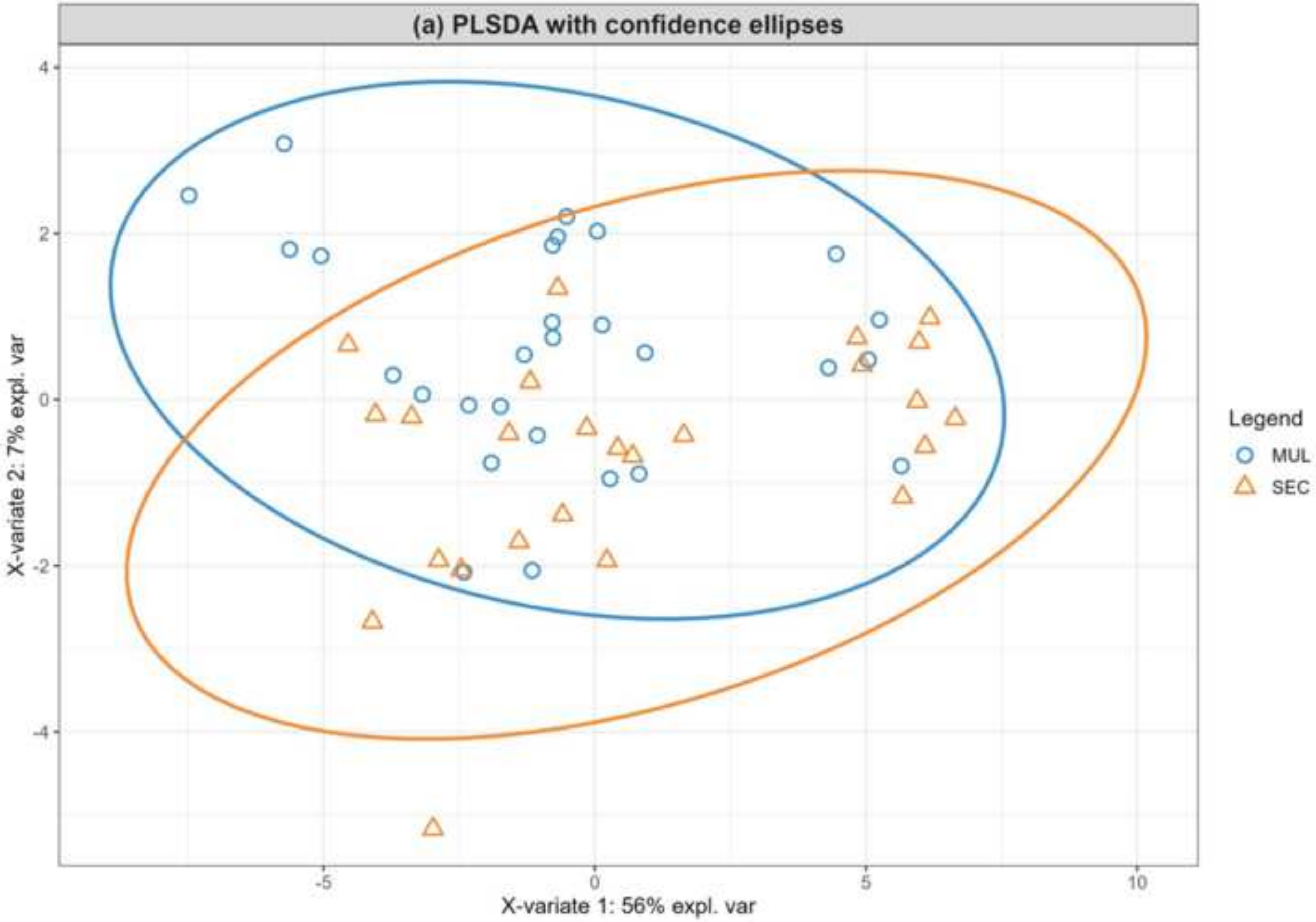

Figure3

Click here to access/download;Figure;Figure3.png

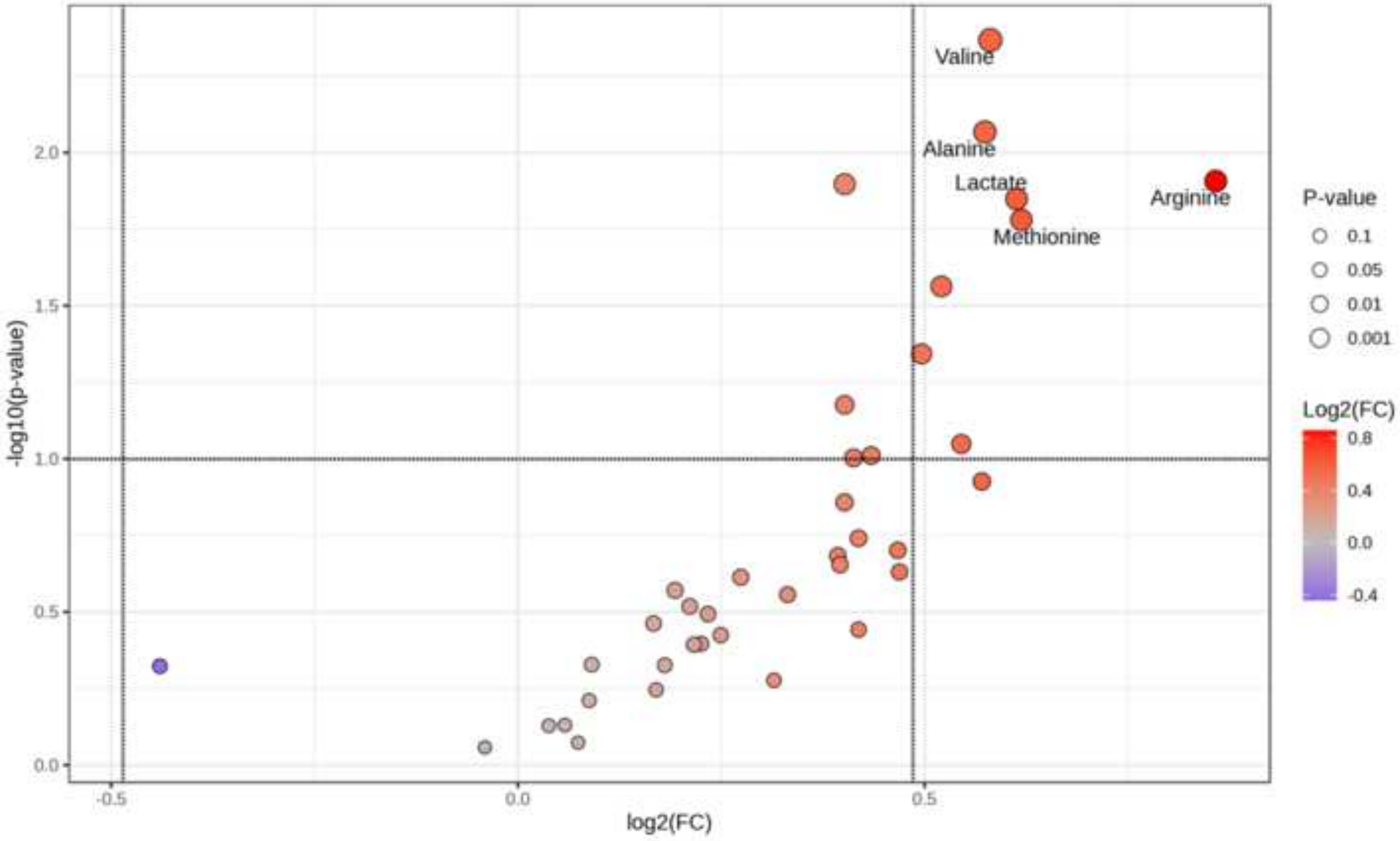

Figure4

Click here to access/download;Figure;Figure4.png

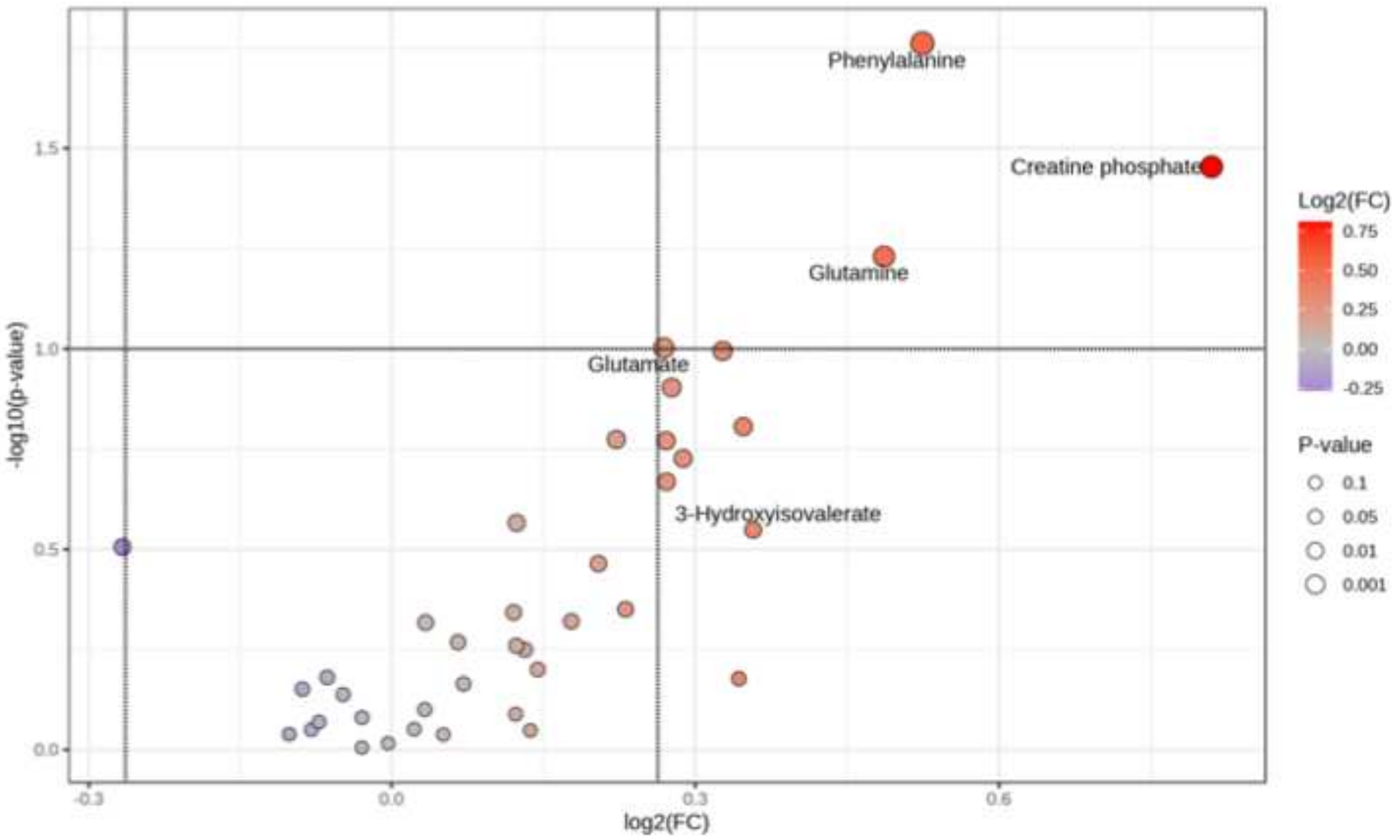

Figure5

Click here to access/download;Figure;Figure5.png

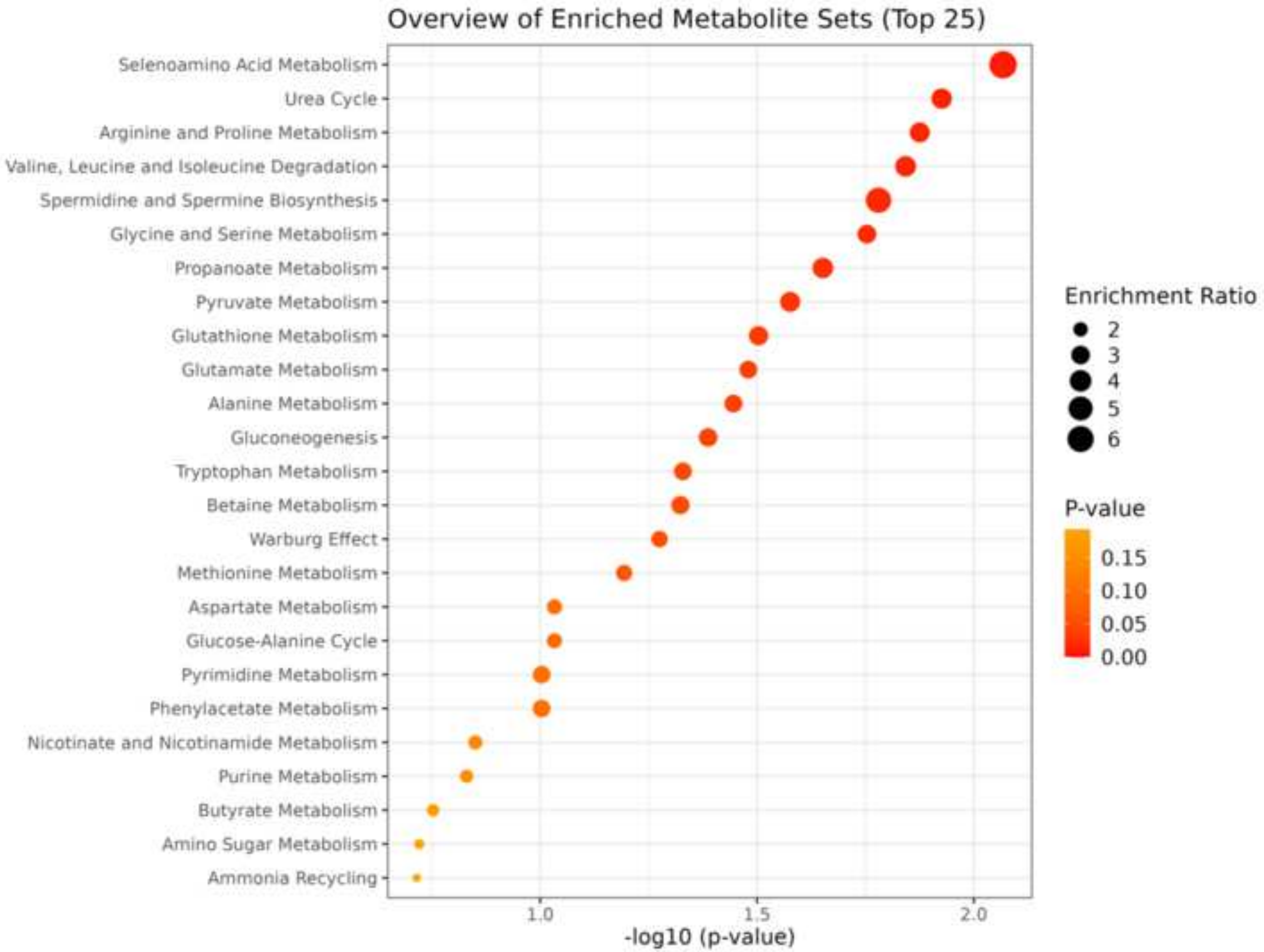

Figure6

Click here to access/download;Figure;Figure6.png

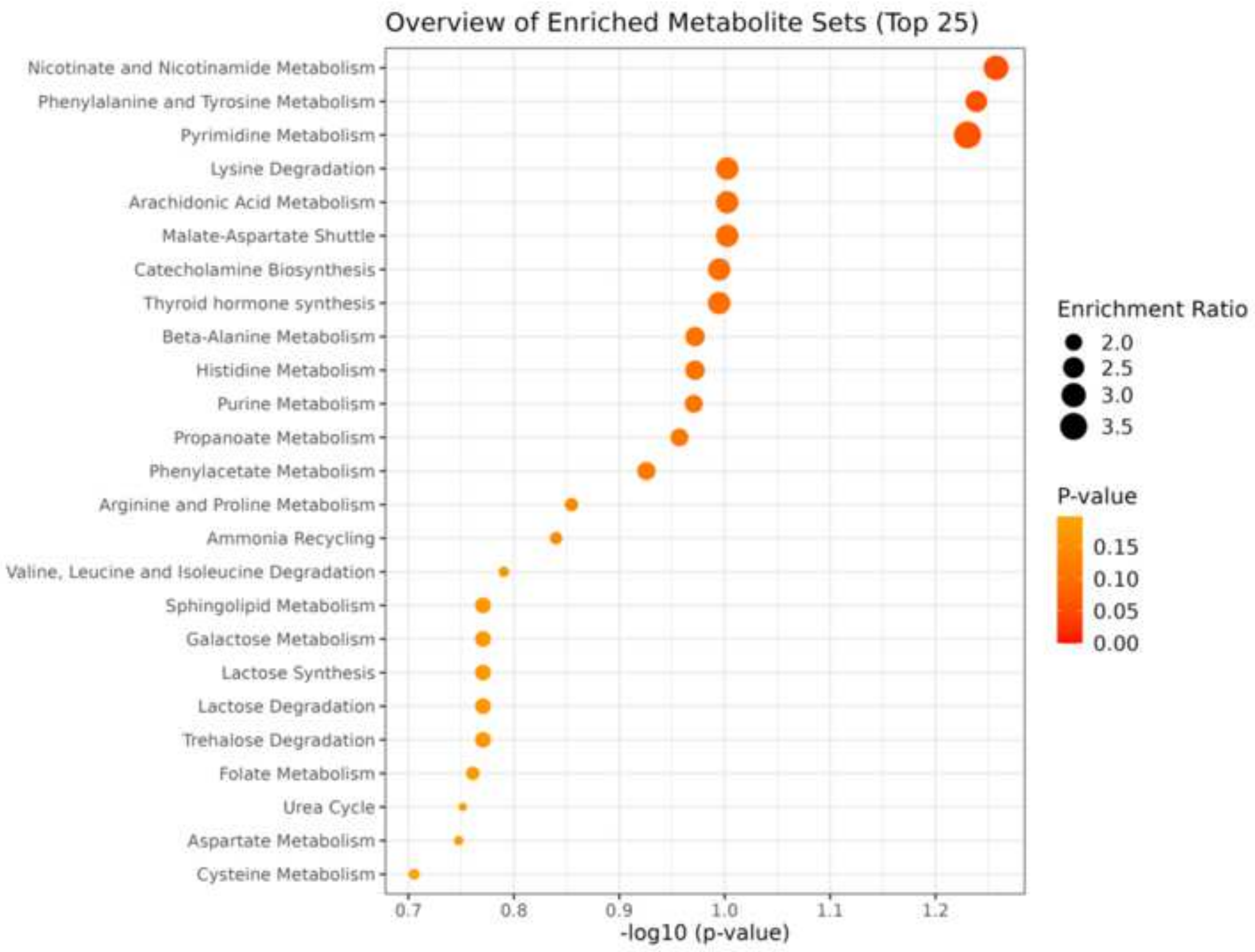

Supplement: Attachment — Submitted filename: PONE-D-25-05801.pdf [file pone.0327152.s005.pdf]
