# Supplementary material for: Fetal programming through early weaning shapes the metabotype of Nelore heifers
Source: PLoS One. 2025 Aug 22;20(8):e0327152. doi: 10.1371/journal.pone.0327152 (PMC12373197; doi:10.1371/journal.pone.0327152)
Supplement: Attachment — Submitted filename: Reviewers response.docx [file pone.0327152.s007.docx]

When submitting your revision, we need you to address these additional requirements.

1. Please ensure that your manuscript meets PLOS ONE's style requirements, including those for file naming. The PLOS ONE style templates can be found at

<https://journals.plos.org/plosone/s/file?id=wjVg/PLOSOne_formatting_sample_main_body.pdf> and

[https://journals.plos.org/plosone/s/file?id=ba62/PLOSOne_formatting_sample_title_authors_affiliations.pdf](https://track.editorialmanager.com/CL0/https:%2F%2Fjournals.plos.org%2Fplosone%2Fs%2Ffile%3Fid=ba62%2FPLOSOne_formatting_sample_title_authors_affiliations.pdf/1/010f01965774d898-7413cd35-efb1-44af-93ff-dd87cc2fde3d-000000/8IcCzBDuUyWBwKasksOFnaGTjh9SOPjoRt4jBYwIbMI=207).

2. We note that this submission includes NMR spectroscopy data. We would recommend that you include the following information in your methods section or as Supporting Information files:

1) The make/source of the NMR instrument used in your study, as well as the magnetic field strength. For each individual experiment, please also list: the nucleus being measured; the sample concentration; the solvent in which the sample is dissolved and if solvent signal suppression was used; the reference standard and the temperature.

Answer: As suggested by the reviewer, the requested information is in the experimental part. In addition, some information described in the experimental part has been added to make the procedures clearer.

2) A list of the chemical shifts for all compounds characterised by NMR spectroscopy, specifying, where relevant: the chemical shift (δ), the multiplicity and the coupling constants (in Hz), for the appropriate nuclei used for assignment.

Answer: As suggested by the reviewer, in the supporting information, a table presenting the chemical shifts, multiplicity and coupling constants for the 1H nucleus of the main characterized metabolites has been added.

3)The full integrated NMR spectrum, clearly labelled with the compound name and chemical structure.

Answer: We appreciate the reviewer’s suggestion. For each 1H NMR spectrum, the signal areas of the identified metabolites in each were integrated using the Chenomx Profiler software. In the experimental part, a Chenomx Profiler file is available with the identified metabolites and the integration region used by the software. Also, compound name and chemical structures are presented in the file.

We also strongly encourage authors to provide primary NMR data files, in particular for new compounds which have not been characterised in the existing literature. Authors should provide the acquisition data, FID files and processing parameters for each experiment, clearly labelled with the compound name and identifier, as well as a structure file for each provided dataset. See our list of recommended repositories here: [https://journals.plos.org/plosone/s/recommended-repositories](https://track.editorialmanager.com/CL0/https:%2F%2Fjournals.plos.org%2Fplosone%2Fs%2Frecommended-repositories/1/010f01965774d898-7413cd35-efb1-44af-93ff-dd87cc2fde3d-000000/W8KgvrfYSj4aykhYbI2xWuPinbKnFdJlC9GSG7AxCpM=207)"

Answer: We appreciate the reviewer’s suggestion. The FIDs of the spectra will be made available on Supplementary table S1. All metabolites identified in the samples are commonly determined in bovine serum samples via NMR spectroscopy.

3.  We note that the grant information you provided in the ‘Funding Information’ and ‘Financial Disclosure’ sections do not match.

When you resubmit, please ensure that you provide the correct grant numbers for the awards you received for your study in the ‘Funding Information’ section.

4. Thank you for stating the following financial disclosure:

[This study was supported by The Coordenação de Aperfeiçoamento de Pessoal de Nível Superior - Brasil (CAPES) - Finance Codes 001, and Fundação de Amparo à Pesquisa do Estado de São Paulo (FAPESP) grant 2020/08845-3, 2017/18937-0, 2022/07906-4, 2005/00509-8

Please state what role the funders took in the study.  If the funders had no role, please state: ""The funders had no role in study design, data collection and analysis, decision to publish, or preparation of the manuscript.""

Author: We have added a paragrapher in the Cover letter

5. In the online submission form, you indicated that [Data can be shared once required to the corresponding author].

All PLOS journals now require all data underlying the findings described in their manuscript to be freely available to other researchers, either 1. In a public repository, 2. Within the manuscript itself, or 3. Uploaded as supplementary information.

This policy applies to all data except where public deposition would breach compliance with the protocol approved by your research ethics board. If your data cannot be made publicly available for ethical or legal reasons (e.g., public availability would compromise patient privacy), please explain your reasons on resubmission and your exemption request will be escalated for approval. "

Please include this amended Role of Funder statement in your cover letter; we will change the online submission form on your behalf.

Author: We have added a paragrapher in the Cover letter. Regarding the Plos Journals policy on data available, we have add a suplementary material with all metabolic data information.

Reviewers' comments:

5. Review Comments to the Author

Please use the space provided to explain your answers to the questions above. You may also include additional comments for the author, including concerns about dual publication, research ethics, or publication ethics. (Please upload your review as an attachment if it exceeds 20,000 characters)

Reviewer #1: Fetal Programming through Early Weaning Shapes the Metabotype of Nelore Heifers is very interesting study for animal nutritionist. However, authors Used “and” and “metabolism “extensively throughout the text. Need to improve sentences. Generally, need to improve English. I will suggest few changes in the manuscript before publication. Highlighted the words need to rewrite. These changes are following: Write full spellings of abbreviations used in abstract.
On line #n 29: Write full spelling of PLS-DA when you are using first time in the text. Please, also consider it for all abbreviations if there is any same case.

Author: Corrected

On line #37: Please, mention plasma or any other organ parameters such as phenylalanine, creatine phosphate, and glutamines?

Author: Corrected

On line # 37: what nutrients availability?

Authors: In this case, we are referring to heifers from multiparous cows as having greater blood nutrient support to aid their growth and development. We have adjusted the sentence to make the information more precise.

What authors will suggest to the animal nutritionist for feed formulation or nutrients requirements for better development considering parity and weaning from this study?

Authors: It is important to emphasize that the heifers evaluated in the present study were weaned at approximately 7 months of age. However, their older siblings were weaned either at the conventional age (7 months) or earlier (5 months). Thus, the heifers in the present study were subjected to fetal programming as a result of the early weaning of their older siblings*.*In this study, all females—whether multiparous or secundiparous—received the same nutrition. However, considering the metabolism of their calves, it might be beneficial to separate calves from multiparous and secundiparous cows for specific nutritional strategies. This could be a good idea. Nevertheless, the aim of this study was to evaluate heifer metabolism according to fetal programming and cow category. For that, there were no observed changes in diet.

Do authors think metabotype results will have same findings in cows weaned on other forages spp rather than Brachiaria spp. Pasture as in this study.

Authors: The diet is a very important factor on animals metabolism and metabotype, so if different nutritional stratagies (forage spp or suplementatuion) has been applied, it could affect the heifres metaboltype.

Secondly, can we apply these results on for this cow Nellore breed or for other cow breeds as well as for sheep/goat etc.
How these results can improve meat quality?

Authors: Unfortunately, based on these results, we cannot suggest that the same would occur with other species, since differences on their metabolism, management and nutrition, but with other cow breed, it could be possible to suggest similarities on th eresults. Further studies with sheep and goats are needed to ensure the results would be similar. Meat quality was not tested in this paper, as our goal was related to reproduction. However, considering the potential for improved nutrient availability in heifers from multiparous cows, there could be benefits for average daily gain and carcass weight. Still, I cannot make specific claims about meat quality. Other authors have suggested that fetal programming could improve meat quality by enhancing myogenesis and marbling.

Reviewer #2: This study investigates the effects of early weaning (EW) on the metabolic programming of Nelore heifers, with a focus on interactions between weaning strategy and dam parity (multiparous vs. secundiparous). The research design is logical, and the data provide valuable insights into optimizing cattle management strategies in tropical regions. The manuscript is well-structured, and the methodology is generally sound. However, certain methodological and interpretative aspects require clarification and refinement to enhance scientific rigor.

Major comments:
1. The manuscript lacks data on feed quality and nutrient intake during the dry season. Including details on feed composition and energy intake would clarify whether "nutritional stress" was experimentally relevant.

Authors: Cows and weaned heifers used in the present study was not under nutritional stress caused, they were raised under tropical conditions and suplementaition, details were presented on the papers below. Important to emphasize that the heifers evaluated in the present study were subjected to fetal programming as a result of the early weaning of their older siblings. But not to differences on dams diet or nutritinal stress

Nishimura TK, Goulart RS, Ramírez-Zamudio GD, da Silva AG, de Paula Carlis MS, Abitante G, et al. Effects of early weaning on productive and reproductive performance of Nelore heifers. J Anim Sci 2023;101. https://doi.org/10.1093/JAS/SKAD379.

Nishimura TK, da Silva AG, Abitante G, Dahlen CR, Goulart RS, Zamudio GDR, et al. Effects of early weaning on the reproductive performance of suckled Nelore cows in the subsequent breeding season. J Anim Sci 2023;101:skad330. https://doi.org/10.1093/JAS/SKAD330.

Nishimura TK, Carlis MS de P, Matos ACD, Feltrin IR, Goulart RS, Zamudio GDR, et al. Can early weaning in the previous lactation of beef cows affect the metabolism and postnatal development of female offspring? 2025. https://doi.org/10.21203/RS.3.RS-5882997/V1.

2. A statistical power analysis or effect size estimation should be included to validate the robustness of group comparisons.
Authors: The power test was included in the Suplementary Figure S1.

3. The study focuses on fetal programming effects induced by early weaning, yet the serum sampling was conducted at 14 months of age rather than immediately post-birth. The authors should clarify the rationale for selecting this specific time point.

Authors: We understand your point of view. However, the aim of this study was to evaluate whether fetal programming influences the metabolotype of heifers before the timed artificial insemination protocol. Therefore, blood samples were collected at the beginning of the reproduction protocol.

Minor comments:
1. There are a few minor grammatical errors and typos that should be corrected to polish the text. For instance, in one sentence: “heifers born to multiparous cows had were heavier from 13 to 15 months age…” – this phrasing is incorrect and can be simplified (perhaps “heifers born to multiparous cows were heavier at 13–15 months of age…”). Similarly, “this data my suggests” should likely be “this data may suggest”, and “gene expresison” should be “gene expression”.

Authors: Corrected

2. Line 58 and 59: the citation style here need to be consistent with other places. These references are also missing in the list.

Authors: Corrected

3. Standardize terms (e.g., "metabotype" vs. "metabolic profile") throughout the text.

Authors: Corrected

4. References 23–24 are listed as "Not Published." Replace with formal citations (e.g., DOI links for preprints) or clarify their status.

Authors: Corrected

5. Aside from Table 1, if the authors have additional data (all 39 metabolites measured), providing a full table (perhaps as a supplement) could be beneficial for transparency. This would let interested readers see the magnitude of differences even for non-significant metabolites.

Authors: Corrected. Supplemnetary Table S2 ws included with all other metabolites information, those that were not presneted in Table 1 in the main text.

Reviewer #3: Thank you for this work. It contains many interesting approaches, especially for beef farmers in nutrient-poor areas, for managing cows optimally and ensuring the good development of their unborn calves. I am sure that these approaches will help to optimize productivity in beef production. I have only a few comments and hope to make the paper stronger with them.

In the discussion, you mentioned the metabolites in part. Can you go into that in more detail? Why did you decide to examine these metabolites in advance? I think, it would make it easier for the reader to understand the selection of the metabolites and enables a better understanding of the publication without having to conduct additional research.

Authors: Thanks for your comments. Mostly, the metabolites discussed in the paper were those that presented statistical differences on the Student’s t-test analysisi or volcano plot. For all analysis, all metabolites were tested, but few of them were significant.

Would you generally recommend early weaning at 5 months for beef cows, or does that only apply to cows calving for the first and second time? What consequences would these results have for the field?

Authors: It can be recommended for any cow to wean the calf at 5 months of age; however, primiparous cows or cows that conceived at the end of the breeding season may benefit more from this strategy. This is because primiparous cows are still growing, and cows that conceived at the end of the breeding season will have less time to recover their body condition before the next breeding season. Cows with lower body condition scores may also benefit from early weaning. However, it is important to provide adequate nutrition to calves weaned at 5 months of age.

Were blood samples also taken from the cows to examine the metabolites? I can imagine that there are also differences between the cows, depending on their parity. What about the animal's individual constitution? Do you think that this also has an influence on fetal programming?

Authors: Despite it could show a very interesting results, we didn’t take blood samples from cows. We oly foucused on heifers. We undertsand that parity, and individual metabolic rates could influence at some extension the fetal programing. However, we keept the animals in the same enviroment, manegent and diet, also using a good number of replicates in order to decrease the individuality effect

A non-publication related question, based on interest: Are there differences in the success of artificial insemination in cows with calves that were weaned earlier? Will this be presented in further publications? Here, too, it may be expected that there would be a difference between the individual groups (EW, CW) and the parity of the dam. Considering that primiparious cows need longer to recover from calving and used their energy for there own growing, could this also be a consequence of later insemination in the next breeding season? This would also be important information for practitioners to improve the productivity in beef cattle production. I think it would be very important to address this issue in a follow-up paper.

Authors: That is a very nice point. Indeed the data presented here is aly one part of a bigger project. More information could be acess on th eNishimura’s paper below:

Nishimura TK, Goulart RS, Ramírez-Zamudio GD, da Silva AG, de Paula Carlis MS, Abitante G, et al. Effects of early weaning on productive and reproductive performance of Nelore heifers. J Anim Sci 2023;101. https://doi.org/10.1093/JAS/SKAD379.

Nishimura TK, da Silva AG, Abitante G, Dahlen CR, Goulart RS, Zamudio GDR, et al. Effects of early weaning on the reproductive performance of suckled Nelore cows in the subsequent breeding season. J Anim Sci 2023;101:skad330. https://doi.org/10.1093/JAS/SKAD330.

Nishimura TK, Carlis MS de P, Matos ACD, Feltrin IR, Goulart RS, Zamudio GDR, et al. Can early weaning in the previous lactation of beef cows affect the metabolism and postnatal development of female offspring? 2025. https://doi.org/10.21203/RS.3.RS-5882997/V1.

Reviewer #4: Fetal Programming through Early Weaning Shapes the Metabotype of Nelore Heifers

General Assessment
This manuscript presents a well-designed study that explores the impact of fetal programming, induced by early weaning of the previous calf, on the metabolic profile (metabotype) of Nelore heifers. The research addresses a relevant and underexplored question in beef cattle production systems, particularly in tropical environments where nutritional challenges prevail during the dry season. The use of ¹H-NMR metabolomics combined with a factorial experimental design provides robust insights into how early maternal management strategies can influence offspring physiology. The study is clearly written, methodologically sound, and provides novel data that may be valuable for improving cow-calf management and reproductive efficiency in Bos indicus systems.
Nonetheless, some revisions are required before the manuscript can be considered for publication. These revisions include improving the clarity and precision of specific terminology, ensuring compliance with PLOS ONE policies regarding data and funding disclosures, and strengthening the discussion on the biological relevance of key metabolites.

Major Comments
**Terminology Consistency – Use of "Secundiparous"**
The manuscript frequently uses the term “secundiparous,” which is uncommon and may cause confusion. It is recommended to replace this term throughout the manuscript with “second-parity” or “second-calving” cows, which are more standard in the scientific literature.

Authors: Corrected

• **Hypothesis Statement**
While the hypothesis is presented at the end of the introduction, it would benefit from a more direct formulation. A suggested revision: “We hypothesize that early weaning of the previous calf programs the metabolic profile of heifers differently depending on dam parity.”

Authors: Corrected

• **Interpretation of Biological Relevance**
Although key metabolites (e.g., glutamate, acetate, hippurate) are statistically different, their specific roles in pubertal development or reproductive physiology are not always clearly linked in the discussion. The authors are encouraged to expand on the physiological implications of these differences, particularly how they might translate into functional outcomes such as fertility, immunity, or growth performance under commercial conditions.

Authors: Corrected, we have improved the discussion.

• **Conclusion Enhancement**
The conclusions are well supported by the data but could be expanded to include practical implications. For example, how might early weaning strategies be integrated into cow-calf management programs under tropical forage systems?

Authors: One sentence was included in the conclusion.

Minor Comments
• **Table 1 Formatting**
Add a full descriptive title and define the superscript letters (“a”, “b”) clearly in the footnote. Units (mmol) should also be clearly labeled in the column headers.
Authors: Corrected

• **Figures**
Ensure all figures (1–6) are provided in high resolution with stand-alone legends. Each figure should be interpretable without reference to the main text, as per PLOS ONE’s guidelines.
Authors: Corrected

Compliance with Journal Policies
• **Financial Disclosure Statement**
The authors must revise the financial disclosure to align with PLOS ONE policy. Specifically, identify which authors received which grants, and include URLs to the funding agencies. Also, confirm whether the funders had any role in the study design or data interpretation.
Authors: A sentence about it was included in th ecover letter

Final Recommendation
**Recommendation**
This is an important and well-executed study that offers meaningful insights into fetal programming in beef cattle. After addressing the points above, especially the mandatory compliance items related to journal policy, the manuscript will be suitable for publication.
